# Brain network analysis in Alzheimer’s disease and mild cognitive impairment using high-density diffuse optical tomography

**DOI:** 10.1162/IMAG.a.1208

**Published:** 2026-04-24

**Authors:** Emilia Butters, Liam Collins-Jones, Rickson C. Mesquita, Deepshikha Acharya, Elizabeth McKiernan, Axel A.S. Laurell, Audrey Low, Sruthi Srinivasan, John T. O’Brien, Li Su, Gemma Bale

**Affiliations:** Department of Engineering, University of Cambridge, Cambridge, United Kingdom; Department of Psychiatry, University of Cambridge School of Clinical Medicine, Cambridge, United Kingdom; Milner Therapeutics Institute, University of Cambridge, Cambridge, United Kingdom; School of Computer Science, University of Birmingham, Birmingham, United Kingdom; Department of Radiology, Mayo Clinic, Rochester, MN, United States; Sheffield Institute for Translational Neuroscience, University of Sheffield, Sheffield, United Kingdom; Department of Physics, University of Cambridge, Cambridge, United Kingdom

**Keywords:** dementia, Alzheimer’s disease, mild cognitive impairment, near-infrared spectroscopy, optical imaging

## Abstract

Dementia is associated with altered resting-state connectivity, measures of which could aid in its early detection and monitoring. High-density diffuse optical tomography (HD-DOT) is well suited to detect these alterations at scale due to its numerous practical advantages, but it has not yet been applied to dementia. In this study, we investigated resting-state functional connectivity across the prefrontal cortex in individuals with mild cognitive impairment (MCI, *n* = 22), Alzheimer’s disease (AD, *n* = 21), and in healthy controls (*n* = 22). A graph theoretical approach was taken to characterise both global and local patterns of prefrontal connectivity over a 5-minute resting period. We found that individuals with MCI exhibited denser and stronger networks with shorter path lengths, which normalised in AD, accompanied by a redistribution of network hubs that were less stable. These results perhaps reflect the recruitment of additional connections in the early stages of pathology to maintain short-term network stability, which is ultimately associated with less efficient and more fragmented network organisation in later stages. Following the demonstration of HD-DOT’s capacity to detect differences between healthy ageing and AD-type cognitive impairment, this work opens up new possibilities for the use of optical imaging in the study of this clinical population and HD-DOT’s potential for scalable clinical use.

## 1 Introduction

Dementia is a syndrome which encompasses a range of cognitive symptoms, including problems with memory, executive function, and language, which lead to functional impairment (Arvanitakis et al., 2019). The predominant cause of neurodegenerative dementia is *Alzheimer’s Disease* (AD) which is typified by progressive medial temporal lobe (MTL) atrophy and amyloid-*β* and tau pathology exhibiting distinct spatial distributions across the brain (Pereira et al., 2019). Prior to the marked cognitive impairment characteristic of dementia, individuals experience a period of early cognitive decline known as *Mild Cognitive Impairment* (MCI). This MCI stage is thought to be critical for intervention as it represents a “break point” between the ability to compensate for pathological changes and irreversible functional impairment (Østergaard et al., 2012).

The formal diagnosis of dementia or MCI typically requires scoring below a pre-defined threshold on standardised cognitive tests, indicating a departure from what is considered to be “healthy cognitive ageing.” Yet, results on these tests can be highly variable between individuals—they can be influenced by age, education level, and the clinician’s interpretation (Mitchell, 2015)—and how exactly they relate to underlying pathology and disease progression remains unclear. Much effort has been expended to develop a more consistent and objective measure of disease stage primarily through the use of neuroimaging, fluid markers, and genetic testing (Ahmed et al., 2014). In particular, there is considerable focus on the development of biomarkers that rely on tools that are low-cost, accessible, and easily deployable for widespread clinical use. Such a biomarker, or series of biomarkers, could facilitate early recognition and intervention for those at risk of further cognitive decline and dementia. Early intervention is crucial as disease-modifying drugs appear to be most effective early in the disease course (Van Dyck et al., 2023).

The resting-state, or “task-free” condition, is a promising target for biomarker development due to its simplicity and the absence of performance-based confounds. During the resting state, several brain regions exhibit temporally coordinated low-frequency spontaneous neural oscillations referred to as *functional connectivity*. Consistent patterns of these correlations have led to the identification of distinct resting-state networks in the brain (Damoiseaux et al., 2006). Disease-specific alterations in the behaviour of these networks may offer markers for the detection of dementia and provide insights into the early functional changes that may precede later structural changes. Previous studies using functional magnetic resonance imaging (fMRI) have identified altered functional connectivity in AD, primarily in the default mode network (DMN), alongside evidence of a compensatory response characterised by increased connectivity in other networks, such as the ventral attention network (Ibrahim et al., 2021; Li et al., 2014; Vemuri et al., 2012). Similar alterations have been identified in MCI (Binnewijzend et al., 2012) and even in individuals with risk factors for AD but with no cognitive symptoms (Filippini et al., 2009).

Functional connectivity can be represented as a graph structure, in which brain regions are treated as nodes and their interactions as edges, allowing the formal characterisation of connectivity patterns. Studies applying this approach to fMRI data in AD have reported disruptions in network organisation including alterations in small world topology (Supekar et al., 2008) such as changes in clustering coefficients (Brier et al., 2013; Dai et al., 2018; Liu et al., 2012), lower modularity (Brier et al., 2013; Sanz-Arigita et al., 2010), and altered path length (Liu et al., 2012; Sanz-Arigita et al., 2010). While some studies have reported reduced clustering and an overall loss of small worldness in AD (Brier et al., 2013; Dai et al., 2018; Sanz-Arigita et al., 2010; Zhao et al., 2012), others have observed increased local clustering alongside reduced global efficiency (Yao et al., 2010; Zhao et al., 2012). These seemingly divergent findings may reflect different disease severities or different methodological perspectives of the same underlying disruption: a shift away from the optimal balance between segregation and integration that characterises healthy brain networks (He et al., 2009; Vecchio et al., 2024). In MCI, results are variable, with mixed evidence for clustering coefficients (Seo et al., 2013), although several studies have described a reduction in small-world organisation (Khodadadi Arpanahi et al., 2025; Yao et al., 2010). Additionally, research using electroencephalography (EEG) has shown a general “slowing” of electrophysiological signals and reduced signal complexity and synchrony in AD (e.g., Aoki et al., 2023; Zheng et al., 2023). However, while fMRI and EEG are well established and widely adopted in research and clinical practice, their deployment in certain contexts, such as non-clinical environments or low-resource settings, may be constrained by infrastructure requirements, costs, and the need for specialised technical expertise.

Near-infrared spectroscopy (NIRS) is an imaging method that detects changes in cortical oxygenation by measuring relative concentration changes of oxygenated (HbO) and deoxygenated haemoglobin (HbR). As NIRS captures the haemodynamic response, it provides an indirect marker of functional brain activation (though it is important to note that these signals can be influenced by underlying vascular dynamics). High-density diffuse optical tomography (HD-DOT) is a method that builds upon the principles of NIRS. In HD-DOT, a high-density array of sources and detectors is used to collect optical data. Unlike traditional NIRS which operates in the channel space, the optical data are then combined with an MRI-derived anatomical head model to reconstruct three-dimensional maps of cortical oxygenation. These maps can then be used to quantify functional connectivity between brain regions, just as with fMRI. Previous work has found that connectivity measured by HD-DOT is well correlated with that measured by fMRI (Eggebrecht et al., 2014) and graph theory metrics have been shown to be reliable in both low-density NIRS (Niu et al., 2012; Novi et al., 2016) and HD-DOT (Uchitel et al., 2022). But unlike fMRI, HD-DOT is low-cost, wearable, silent, portable, easy-to-use, and relatively tolerant of head motion (Pinti et al., 2018). HD-DOT’s relative accessibility and practicality make it possible to study the brain outside of the noisy, restrictive environments associated with methods such as fMRI, enabling scanning at the bedside, in the home, and with naturalistic experimental paradigms. As HD-DOT also measures two chromophores as opposed to fMRI’s sole measurement of HbR, it consequently offers a promising alternative that combines high spatial and temporal resolution with the portability and scalability needed for widespread clinical use (Butters et al., 2023). HD-DOT is thus an attractive candidate for application to dementia.

To investigate whether HD-DOT can detect alterations in dementia, we examine prefrontal resting-state functional connectivity in individuals with AD-type dementia, AD-type MCI, and in age-matched healthy controls. To our knowledge, this is the first study to apply HD-DOT to these populations. We use graph theory to characterise and quantify patterns of functional connectivity, and then examine the relationship between graph theory-based measures, and severity of cognitive impairment and MTL atrophy. According to the literature, we expect to observe disrupted functional connectivity in AD with less pronounced network perturbation in MCI. Specifically, we anticipate to observe reduced network efficiency, density, and strength in both clinical groups, with the extent of these changes correlating with the severity of cognitive impairment and MTL atrophy.

## 2 Materials and Methods

### 2.1 Participants

A total of 65 subjects took part in this study. Of these, 21 were diagnosed with AD, 22 were diagnosed with MCI, and 22 were age-matched healthy controls (HC). All subjects in the clinical groups had received a formal diagnosis from a memory clinic and fulfilled the diagnostic criteria for either AD-type dementia (McKhann et al., 2011) or AD-type MCI (Albert et al., 2011). Healthy controls were recruited from the community through flyers, recruitment websites, and the families of study participants. To be included as a control, subjects had to have no history of memory problems and scored above 26 on the Mini-Mental State Examination (MMSE, Folstein et al., 1975). Across all groups, subjects were excluded if they had a condition known, or suspected, to affect cerebral blood flow or haemodynamics. This included individuals with a history of vascular events (e.g., stroke or transient ischaemic attack) or a diagnosed respiratory illness (e.g., asthma or chronic obstructive pulmonary disease). All subjects underwent a clinical screening interview and neuropsychological testing which included the MMSE, Montreal Cognitive Assessment (MoCA, Nasreddine et al., 2005), and additional scales covering neuropsychiatric symptoms and functional impairment (Supplementary Table S1). Medial temporal atrophy (MTA) was also scored from 0 to 4 for each subject to provide a brain-specific and widely-used measure of disease severity (Scheltens et al., 1992). Scans were scored upon visual inspection by three independent reviewers who had attended a 2-hour training course but were not expert reviewers, for example, radiologists. The final score was calculated as the sum of the average of their ratings for the left and right hippocampi. The data used in the present work are a subset of that collected as part of the “Optical Neuroimaging and Cognition” study (IRAS ID 319284). This study was approved by Wales Research Ethics Committee. Written informed consent was obtained in person from all subjects, and from informants for subjects in AD or MCI groups.

### 2.2 Experimental paradigm and recording

HD-DOT data were collected during a 5-minute resting-state period using the LUMO (Gowerlabs Ltd., London, UK). This duration was chosen following piloting to minimise the risk of subjects falling asleep. The LUMO is a high-density modular system composed of multi-distance, overlapping NIRS channels. This enables the recording of data from short channels (<12 mm), which we assume sample the scalp, and long channels (>12 mm), which we assume also sample the cortex. Each module contains three dual-wavelength LED sources (735 and 850 nm) and four photodiode detectors. Data were recorded at 12.5 Hz from 12 modules covering the bilateral frontal cortex for a total coverage of 36 sources and 48 detectors (~1728 possible channels). An appropriately sized cap (54–56 cm, 56–58 cm, or 58–60 cm) was selected by measuring subjects’ head circumference prior to fitting.

**Appendix Fig. A1.**
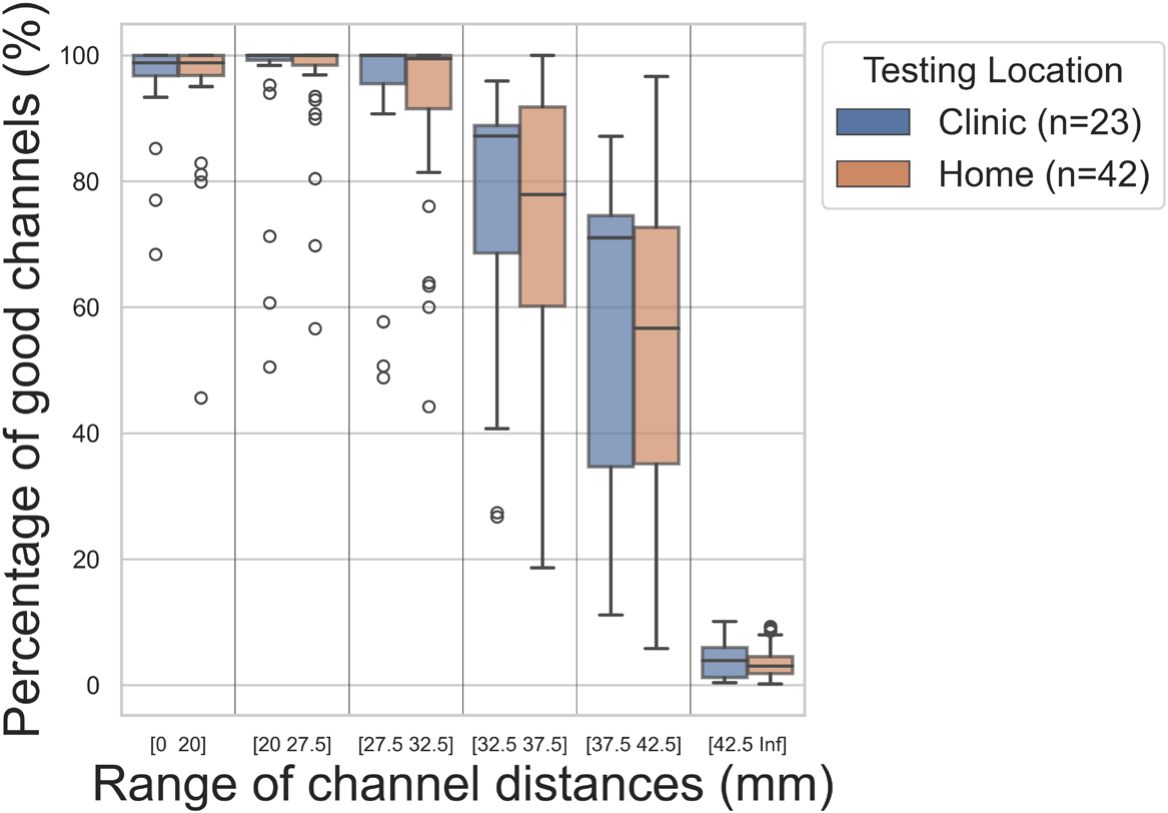
No significant differences in the percentage of good channels across channel distances between testing locations.

For the majority of subjects (67.1%), data collection took place in their homes. For the remainder of subjects, data collection took place in a clinic room at the University of Cambridge. There were no significant differences in the proportion of individuals who were tested at home across groups (*χ*^2^ = 3.41, *p* = 0.18, HC: 50%, MCI: 68%, AD: 76%). Although testing locations varied, all recordings were carried out in quiet, dimly-lit environments. During the recording, subjects were seated in a chair and instructed to rest quietly with their eyes closed while remaining awake, minimising head movements beyond small adjustments, and avoiding structured thoughts or tasks. A comparison of data quality, defined as the percentage of good channels per source–detector distance range, revealed no significant differences between the home and clinic settings (Appendix Fig. A1). During acquisition, a software error in the data-saving process resulted in three datasets being corrupted. These were subsequently re-recorded.

### 2.3 Data pre-processing

The optical data were pre-processed using the Homer2 toolbox (Huppert et al., 2009) in Matlab v2021b (The MathsWorks Inc.; MA, USA). Channels were removed if (1) their mean coefficient of variation was above 8.3% (equivalent to a signal-to-noise ratio <12), (2) their mean signal intensity >1 × 10^11^ V, (3) their source–detector separation >100 mm, as per Uchitel et al., 2022, or (4) heart rate could not be detected (i.e., if the maximum of the Fast Fourier Transform (Cooley et al., 1969) of that channel was not in the 0.5–2 Hz range). Raw intensity signals were first converted to changes in optical densities (Δ*OD*) using Homer2. The proportion of each subject’s recording affected by motion was assessed for motion artefacts using hmrMotionArtifact from Homer2 using an amplitude threshold of 0.5 and a standard deviation of 10, set by default in the DOTHUB toolbox (www.github.com/DOT-HUB/DOT-HUB_toolbox). The median motion burden was 15.3% [8.9–29.6] which is within the acceptable range for HD-DOT (e.g., Srinivasan et al., 2024) so no motion correction was performed to avoid introducing artificial changes into the haemodynamic signal. No significant difference in motion burden was found between groups (*H* = 0.54, *p* = 0.76, HC: 13.1, MCI: 21.4, AD: 17.6). To further assess the potential influence of motion on connectivity estimates, the distance of connections was plotted against the correlation between connection strength and motion burden (QC–FC correlation; Ciric et al., 2017), shown in Supplementary Figure S4. The Δ*OD* data were then filtered using a third-order Butterworth bandpass filter (0.01–0.1 Hz). Potential contamination of the data by scalp haemodynamics was minimised by regressing the signal from the nearest short-separation channel (<12 mm) onto each corresponding long-separation channel using the DOTHUB toolbox.

### 2.4 Source localisation

**Fig. 1.**
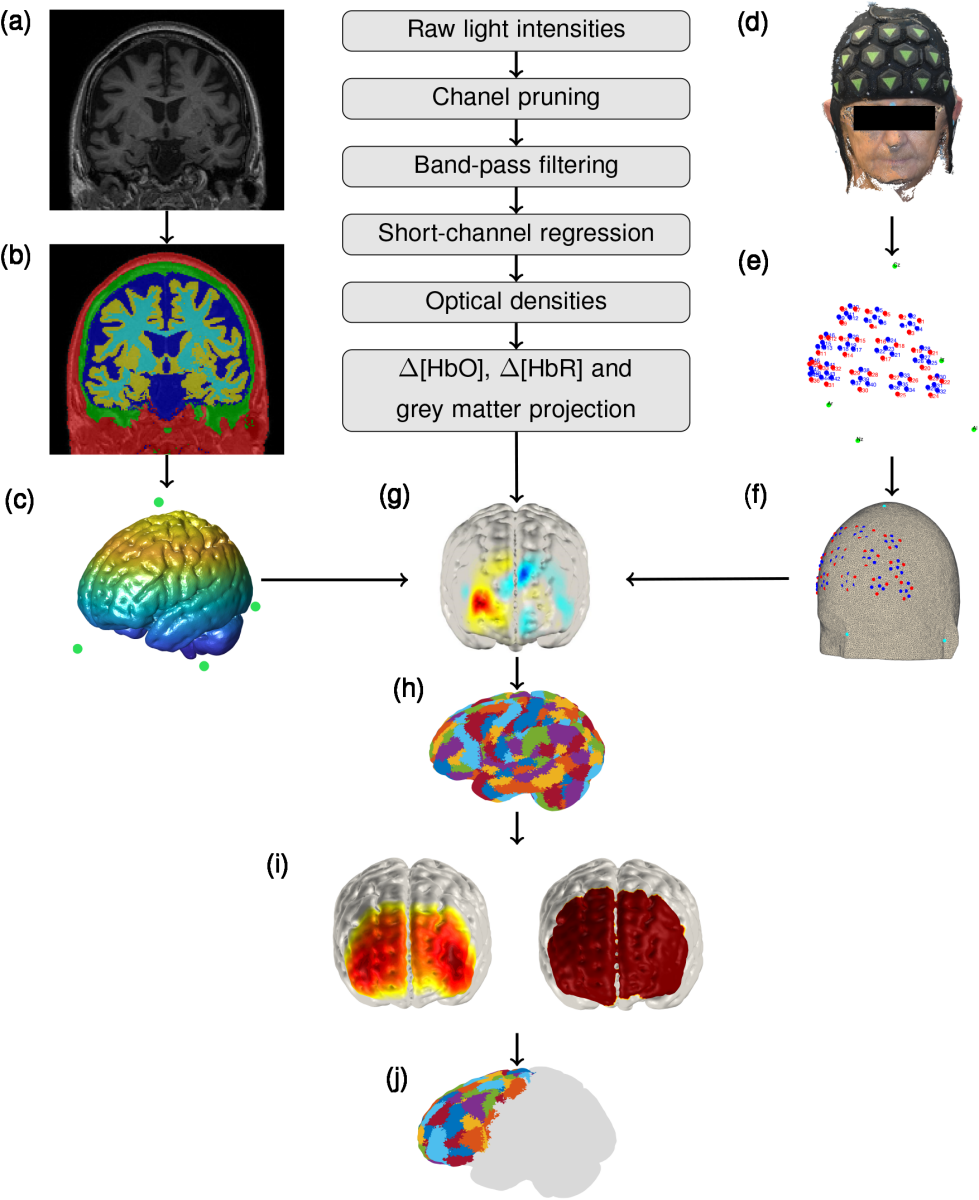
Processing pipeline for the HD-DOT data. (a) An example structural T1-weighted image acquired for subjects in clinical groups. The MNI atlas was used for healthy controls. (b) Segmentation of the structural image into five tissue types using SPM12: scalp, skull, cerebrospinal fluid, white matter, and grey matter. (c) MRI-derived tetrahedral grey matter mesh and cranial landmarks. (d) An example point cloud of a subject wearing the LUMO cap, created using photogrammetry. (e) Manually-extracted optode and cranial landmark coordinates. (f) Optode and cranial landmark coordinates registered to a subject’s tetrahedral mesh. (g) An example topographic reconstruction of cortical oxygenation. (h) Schaefer parcellation atlas registered to native space via non-linear transformation. (i) Sensitivity mask (right) including only nodes sensitive to >5% of the normalised Jacobian (left). (j) Only parcels with >50% of sensitive nodes included per subject. Time series of each included node averaged per parcel.

As head size and shape vary across the population, we cannot assume that the same source–detector pair will sample exactly the same brain region in different subjects. To account for this, photogrammetry was used to digitise the locations of the optodes and cranial landmarks for each subject (as per Vidal-Rosas et al., 2021). This method involves aligning the features from multiple photographs to create a point cloud from which the coordinates of each desired point can then be extracted. To facilitate this, bright green equilateral triangles (~18 mm length per side) were placed on each LUMO module such that the vertex of each triangle overlays a source (Fig. 1d). Similarly, bright blue circular stickers (8 mm in diameter) were placed over five cranial landmarks: the left pre-auricular point (Al), the right pre-auricular point (Ar), the nasion (Nz), the inion (Iz), and the vertex (Cz), measured as halfway between Nz and Iz. Each point cloud was generated from two 360° videos of subjects’ heads using Agisoft Metashape (Agisoft LLC, St. Petersburg, Russia). The resulting models were then scaled using MeshLab 2023 (Cignoni et al., 2008) according to known dimensions between sources provided by the manufacturer. The approximate coordinates of each source and detector were manually extracted using a custom script written in Matlab v2021b (Fig. 1e).

### 2.5 Head modelling and registration

To create tomographic maps of brain oxygenation, the high-density NIRS data were reconstructed using a head model. For healthy controls, the Montreal Neurological Institute (MNI)-152 template (Mazziotta et al., 1995) was used, as we assume that these subjects have typical brain anatomy and tissue organisation for which standard atlases are sufficient (Ferradal et al., 2013). However, due to the cortical atrophy that is associated with neurodegenerative dementias (Mouton et al., 1998), subject-specific MRI-derived head models were used for subjects with AD and MCI. As light propagates through different tissue types in distinct ways, using a subject-specific head model enables the light propagation model to account for pathology-related changes in brain size, shape, and tissue boundaries. This ensures that anatomy-driven differences in light sensitivity or signal origin are not misattributed to functional changes in the cortex. To create these head models, structural T1-weighted MRI scans were acquired for each subject with AD or MCI (Fig. 1a; for acquisition details see Appendix Table A1).

Subject-specific head models were created by first bias-field correcting the structural MRIs using FAST from FSL (Woolrich et al., 2008) and then segmenting them using SPM12 (www.fil.ion.ucl.ac.uk/spm) into five tissue types (Fig. 1b): grey matter, white matter, cerebrospinal fluid, skull, and scalp. The segmentations were used to generate voxelised tissue masks for each tissue type which were then combined to create a single labelled map per subject. MRI-derived cranial landmarks were manually extracted using ITK-SNAP (Yushkevich et al., 2006), where Cz was estimated. The labelled tissue map and landmarks were used to construct a three-dimensional tetrahedral mesh using Iso2mesh (Fang & Boas, 2009) in Matlab v2021b (Fig. 1c). The optode positions were then registered to the subject’s native space using an affine transformation between the MRI- and photogrammetry-derived cranial landmarks (Fig. 1f).

### 2.6 Image reconstruction and parcellation

To reconstruct the optical data, a forward model of light propagation was solved using the finite element method (Schweiger et al., 1993), implemented using Toast++ (Schweiger & Arridge, 2014). Light propagation was modelled using the diffusion approximation to the radiative transfer equation (Arridge, 1999). The sensitivity of the optical signals (*S*) to changes in absorption coefficients was then estimated by deriving a Jacobian matrix (*J*) for each wavelength. To resolve for changes in absorption coefficient, the Jacobian was pseudo-inverted via the Moore–Penrose method. A zeroth-order Tikhonov regularisation was applied using a hyperparameter of 0.01 to stabilise the solution. The resulting images were then converted to images of per-node changes in concentration of HbO and HbR (Δ *C*) using the modified Beer-Lambert law (Cope, 1991; Delpy et al., 1988). The forward model, its inversion, and image reconstruction were implemented using the DOTHUB toolbox.

To facilitate region-specific analyses and reduce data dimensionality, the reconstructed images were parcellated using the 400 parcel Schaefer parcellation atlas (Fig. 1h; Schaefer et al., 2017). This granularity was selected based on preliminary testing, as it effectively reduced data size while providing stable and sufficient parcel coverage across subjects for comparing network architectures. In the Schaefer atlas, each node in the grey matter mesh is assigned a functionally-relevant cortical *parcel* (Uchitel et al., 2022). Each parcel in this atlas is also allocated to one of 17 resting-state networks (Yeo et al., 2011). For data reconstructed using a subject-specific head model, the atlas was non-linearly transformed to each subject’s native space using antsRegistration from Advanced Normalisation Tools (Avants et al., 2010) as the atlas is registered to MNI space. Nodes in the grey matter mesh were matched to the closest node in the parcellation atlas using a k-nearest neighbour algorithm. For each subject, a parcel was only included if >50% of nodes in that parcel were sensitive. A node was defined as sensitive if any good-quality channel for that node exhibited a normalised Jacobian sensitivity exceeding 5% of the channel-wise maximum across both wavelengths (Fig. 1i; Uchitel et al., 2022).

A single time series for each parcel was then obtained by averaging the time series of all sensitive nodes within that parcel (Fig. 1j). Only Δ*HbO* was analysed in the present study as it generally has a higher signal-to-noise ratio and statistical power than Δ*HbR* (Tachtsidis I and Scholkmann F., 2016). Global mean signal regression was then applied to the parcel-level HbO time series to mitigate the impact of systemic physiological fluctuations across groups. Although short-channel regression corrects for scalp-level systemic signals, deeper vascular contributions may still influence functional connectivity estimates (Lanka et al., 2022). The results for the functional connectivity analysis without global signal regression are included in the Supplementary Material as Figure S5. To assess the contribution of global systemic-related signal fluctuations, the global component for each subject was correlated with each parcel’s time series, and the mean parcel–global correlation was compared between groups.

### 2.7 Graph theory analysis

**Fig. 2.**
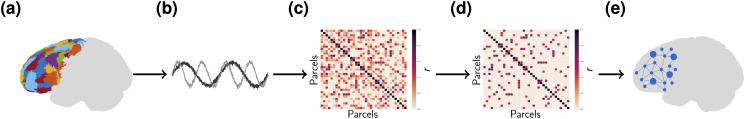
Functional connectivity analysis pipeline. (a) Parcellation of the reconstructed image. (b) Time course extracted from each parcel. (c) Pearson’s *r* correlation matrix calculated for all parcels. (d) Matrix thresholded to remove spurious connections. (e) Graph construction.

Functional connectivity was assessed using a graph theory approach. Networks can be represented by graphs: a set of vertices (*V*) connected by edges (*E*). In the present case, the vertices represent brain regions, that is, parcels, and the edges represent functional connectivity, that is, time-dependent coordinated activity between these regions. For each subject, functional connectivity was calculated by computing the Pearson’s correlation coefficient (*r*) between the time series of each pair of parcels across the entire 5-minute time course (Fig. 2b, c). Accordingly, each connectivity matrix had the size *N* × *N* where *N* is the number of sensitive parcels for that subject.

For graph analysis, a weighted undirected adjacency matrix was created from the pairwise Pearson’s correlation between parcels, shown in Supplementary Figure S3. To do so, correlation matrices were first transformed using Fisher’s r-to-z transform to stabilise variance, then thresholded using an absolute *r* threshold of 0.2 to remove spurious correlations (Novi et al., 2016; Fig. 2d). Edge weights were only retained for edges exceeding this threshold and were assigned their z-transformed values. These values were subsequently re-scaled by dividing by 2.65 to ensure that all values remained below 1, allowing the graph to be interpreted as weights while avoiding divergence as *r* → 1. The resulting matrices were treated as subject-specific graphs for subsequent analyses. Graph sparsity was quantified given its widespread use for thresholding connectivity matrices in network neuroscience. This was done as follows:

$$\text{Sparsity}=1-\text{Density}=1-\frac{2E}{V\left(V-1\right)}$$ (1)

**Fig. 3.**
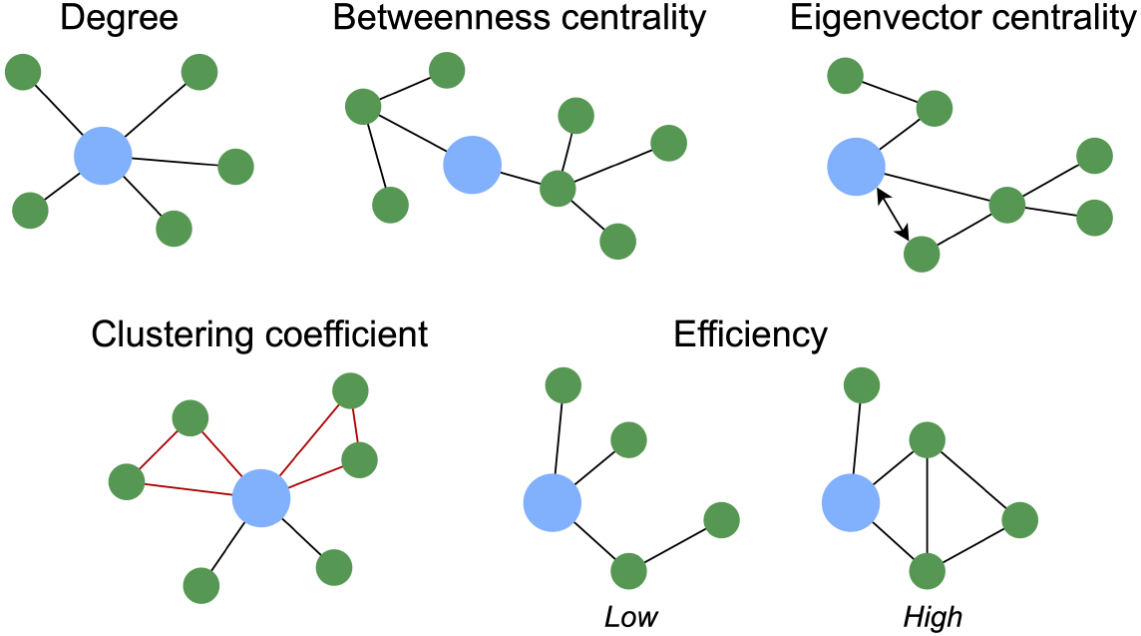
Graph theory metrics. Degree—the number of connections a node has. Betweenness centrality—how important a node is for connecting other nodes. Eigenvector centrality—how well connected a node’s neighbours are. Clustering coefficient—how likely a node’s neighbours are to be connected to each other. Efficiency—how easily information can flow through a network.

Following graph construction for each subject (Fig. 2e), various metrics were calculated to characterise the connectivity patterns of each graph using the Brain Connectivity Toolbox (Rubinov & Sporns, 2010). Connectivity was assessed both globally and locally. Global network connectivity was evaluated across the entire graph for each subject by averaging graph theory metrics across all of a subject’s sensitive parcels (Fig. 3). Connectivity was assessed via degree and centrality (betweenness centrality and Eigenvector centrality), segregation was assessed via clustering coefficient, and flow was assessed via efficiency. Total strength was calculated as the sum of the weights of all edges between nodes in the graph. As head motion can alter connectivity estimates (Van Dijk et al., 2011), the relationship between each graph theory metric and head motion was quantified. No significant associations were observed.

**Fig. 4.**
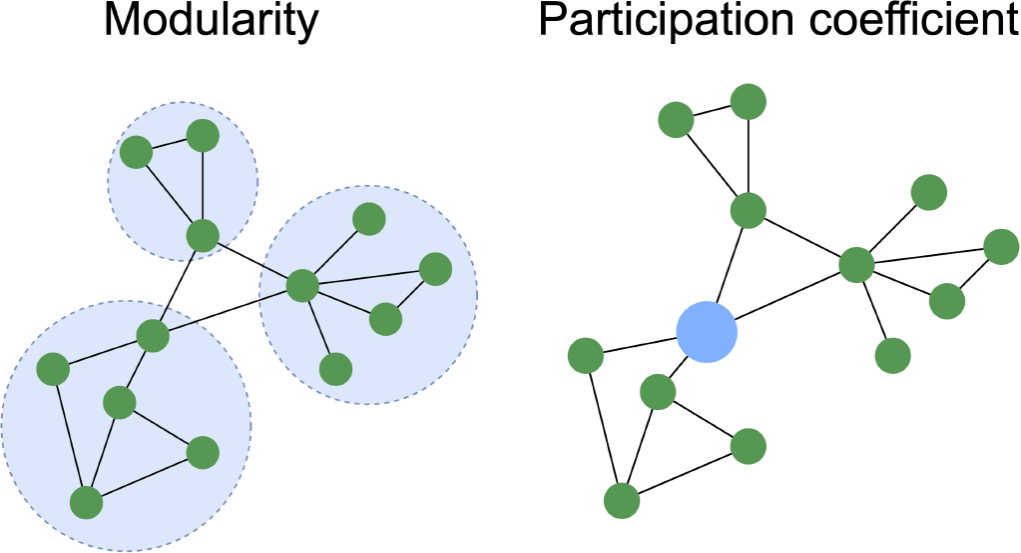
Clustering properties. Modularity—how well a network can be divided into modules. Participation coefficient—how connected a node is to different modules.

Local connectivity was then assessed by concatenating all subjects’ data parcel-wise. This means that analyses were restricted to individual parcels and did not consider edges that spanned the broader network to avoid incorporating global interactions. Clustering properties were first evaluated (Fig. 4). Modularity was calculated using the Louvain algorithm (Blondel et al., 2008) which quantifies the strength of community structure in a network by partitioning it into distinct clusters. Following this, the participation coefficient was computed for each parcel as the proportion of its degree that connects it to modules other than the one it belongs to. Participation coefficients were then averaged per resting-state network (Yeo et al., 2011), excluding networks present in <20% of subjects (i.e., dorsal attention, visual, somatomotor, and temporoparietal). This means that out of the 17 Yeo networks, 10 were included in this analysis.

**Fig. 5.**
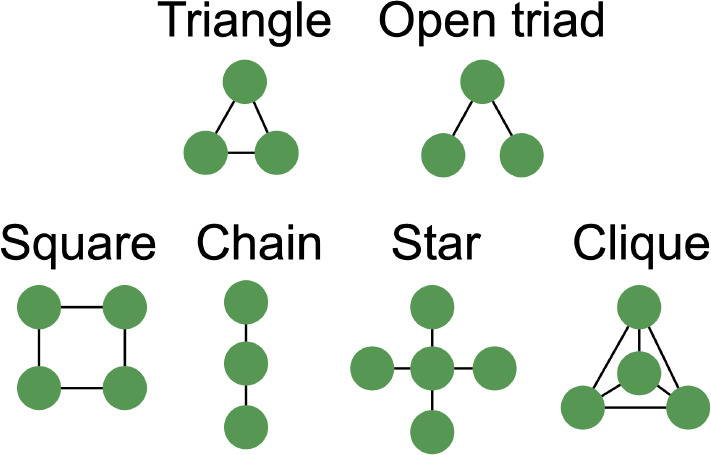
Three-node (top) and four-node motifs (bottom) were considered.

The number of distinct sub-patterns, termed *motifs*, was calculated per graph and subsequently normalised by the total possible number of each motif per graph. Motifs are recurring sub-patterns within a larger network that may reflect functional regularities. The frequency of three-node motifs, triangle and open-triad, and four-node motifs, square, chain, star, and clique, was calculated (Fig. 5).

Finally, to identify important network hubs across groups, the frequency with which parcels were found in the top 10% for participation coefficient, degree, and betweenness, and eigenvector centrality was calculated. This threshold was selected to be consistent with previous approaches in fMRI (Smith et al., 2023) and NIRS (Novi et al., 2016). An aggregate “overall centrality” score was defined as the median of the three. Parcels with low counts (i.e., those identified in <15% of subjects in each group) were removed and only parcels defined as hubs in at least 20% of subjects were thereafter included. Thresholding parcel counts ensured consistency across subjects, and retaining only parcels with the top connections follows standard practice (Garrison et al., 2015). Accordingly, 26 parcels were included for the healthy control group, 33 parcels for the AD group, and 21 parcels for the MCI group. Functional connectomes were visualised using the NetworkX module (Hagberg et al., 2008) in Python v3.8.8.

### 2.8 Statistical analysis

Group-level differences in graph theory metrics, demographic variables, and clinical data were calculated following the removal of missing values (if necessary) and assessment of normality using the Shapiro–Wilk test (Shapiro & Wilk, 1965). Depending on the distribution of the data, either parametric or non-parametric statistical tests were used for all group-level and post hoc analyses. Correlations between global graph theory metrics and age, MMSE score, and MTA rating were determined using a Pearson’s or Spearman’s correlation coefficient. Group differences in sex ratio, and the proportion of subjects with a family history of dementia or stroke were assessed using the chi-squared (*χ*^2^) test. Statistical analyses were conducted using either Python v3.8.8 or Matlab v2021b with significance thresholds set at *p* < 0.05. The Benjamini–Hochberg procedure was used to control the false discovery rate (FDR; Benjamini & Hochberg, 1995) unless otherwise stated.

## 3 Results

### 3.1 Study population summary

A summary of subject characteristics is shown in Table 1. There were no significant differences in age or sex ratio across groups. Full neuropsychological test scores are detailed in Supplementary Table S1.

**Table 1.** Summary of demographic and clinical data across study groups.

	HC (*n* = 22)	MCI (*n* = 22)	AD (*n* = 21)	*p*
Age (y)	74.5 ± 6.16	75.0 ± 6.64	72.6 ± 8.42	0.40
Female (%)	50.0	45.5	42.9	0.89
Education (y)	20.0 ± 3.67	18.7 ± 3.31	17.8 ± 2.93	0.18
Family history of dementia (%)	59.1	68.2	66.7	0.80
Family history of stroke (%)	27.3	22.7	28.6	0.90
MTA rating	n/a^*^	3.36 ± 1.8	4.90 ± 1.50	**0.01**
MMSE score	28.8 ± 1.15	26.5 ± 2.23	21.3 ± 4.35	**0.00** ^a,b,c^
MoCA score	26.1 ± 2.07	23.2 ± 3.45	16.3 ± 5.23	**0.00** ^a,b,c^

Shown as mean ± standard deviation. *P*-value indicates overall group-level comparison. Bold denotes differences significant at a group level. Superscript letters denote significant pairwise group comparisons: ^a^ HC vs MCI, ^b^ HC vs AD, ^c^ MCI vs AD. ^*^ MRIs were not acquired for healthy controls so MTA was not rated. MTA rating shown as the sum of left and right hemispheres.

### 3.2 Functional connectivity

**Fig. 6.**
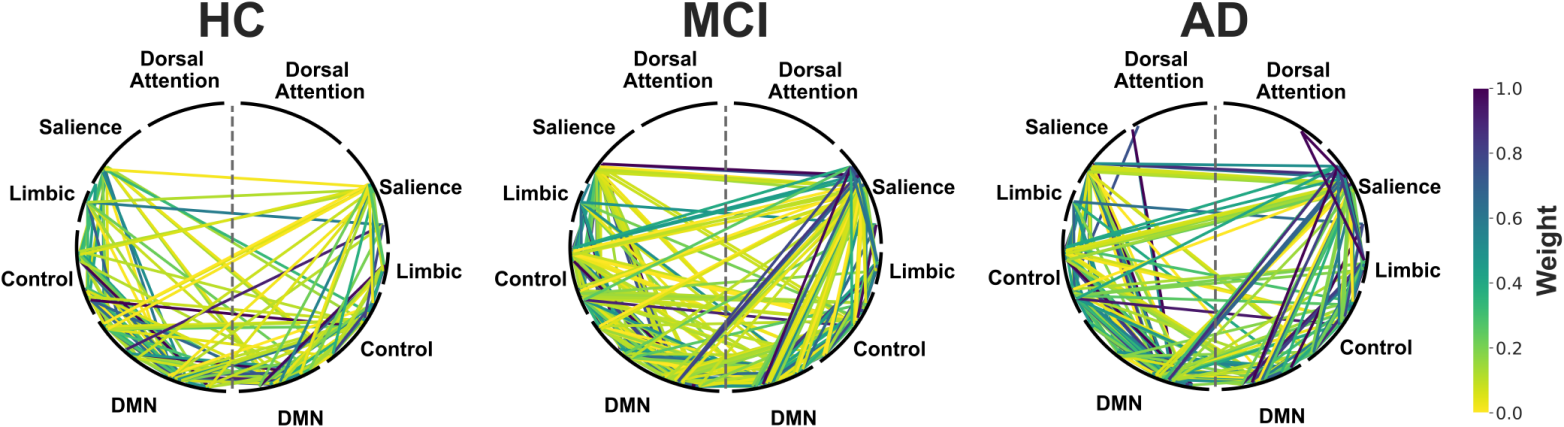
Functional connectomes showing significant connections (*p* < 0.05) present in healthy controls, mild cognitive impairment, and Alzheimer’s disease. Shown as weighted edges. Thresholded to only include significant connections *p* < 0.05 present in >20% of subjects and spurious connections (*r* < 0.2) removed. Parcels grouped according to resting-state network (Yeo et al., 2011). The visual, temporo-parietal, and somato-motor networks are not shown as these did not contain any sensitive parcels. A grey dashed line divides left and right hemispheres. DMN = default mode network.

The functional connectomes for each group are shown in Figure 6. Graph sparsity was significantly higher for the MCI group than for healthy controls (*g* = 0.92) and the AD group (*g* = 0.89; *p*_FDR_ < .05). Global component coupling to parcel time series was higher in both MCI (*g* = 0.74, *p*_FDR_ < .05) and AD (*g* = 1.00, *p*_FDR_ < .01) than in healthy controls.

#### 3.2.1 Global dynamics

**Fig. 7.**
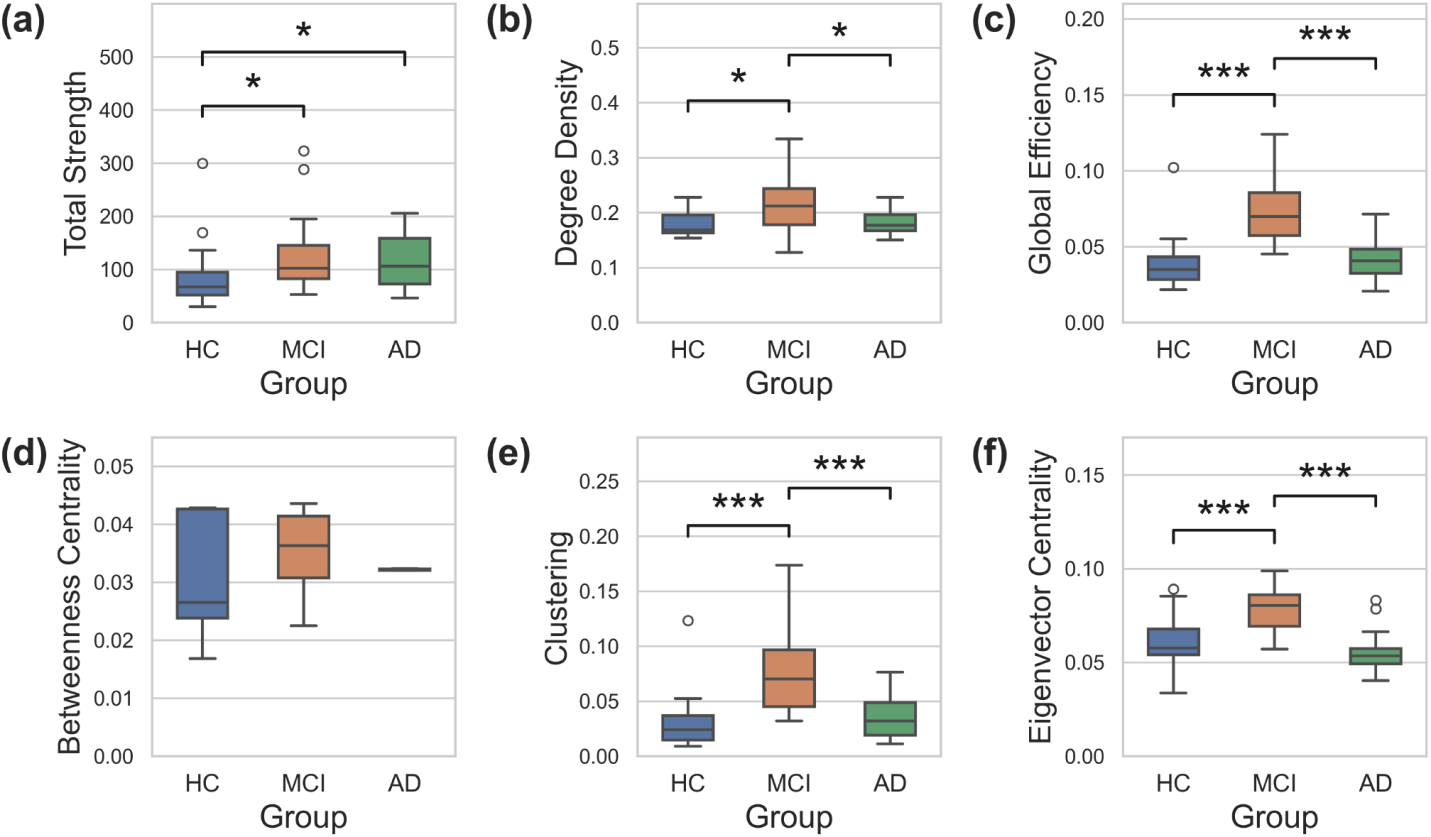
Group-level global functional connectivity, reported as the median with interquartile range: (a) total strength, (b) degree density, (c) global efficiency, (d) betweenness centrality, (e) clustering, and (f) eigenvector centrality. Statistical significance demonstrated by *, *p* < 0.05; **, *p* < 0.01; ***, *p* < 0.001; FDR corrected.

**Appendix Fig. A2.**
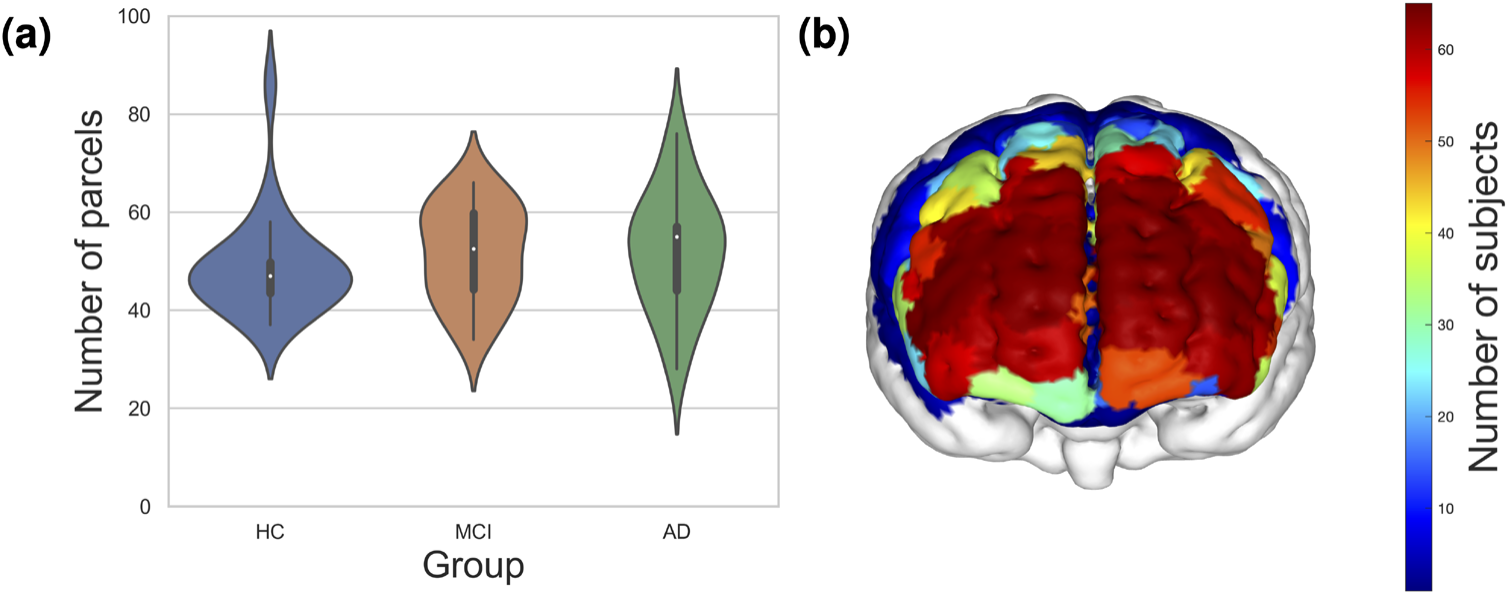
Coverage of the frontal array. (a) The number of sensitive parcels for each subject per group. The median number of sensitive parcels was 55 for the AD group, 52.5 for the MCI group, and 47 for the healthy control group. (b) Histogram of the number of subjects sensitive to all frontal parcels.

Significant differences in global functional connectivity were found across several graph theory metrics between healthy controls and both clinical groups, as well as between MCI and AD groups. Specifically, total strength was higher in AD (*g* = 0.56, *p*_FDR_ < .05; Fig. 7a) and MCI (*g* = 0.64, *p*_FDR_ < .05) than in healthy controls. A pattern of increased connectivity in MCI compared with both healthy controls and the AD group was observed for degree density (HC: *g* = 0.75, AD: *g* = 0.71; *p*_FDR_ < .05; Fig. 7b), global efficiency (HC: *g* = 1.81, AD: *g* = 1.80; *p*_FDR_ < .001; Fig. 7c), clustering (HC: *g* = 1.41, AD: *g* = 1.35; *p*_FDR_ < .001; Fig. 7e), and eigenvector centrality (HC: *g* = 1.39, AD: *g* = 2.02; *p*_FDR_ < .001; Fig. 7f). Although several of the graph theory metrics scale with the number of parcels present (which may introduce bias between groups), no significant differences were found between the number of sensitive parcels across groups (*H*(2) = 2.10, *p* = 0.35; Appendix Fig. A2a; Supplementary Figs. S1 and S2). In sum, these results indicate *increased* connectivity in MCI, characterised by denser networks, shorter pathlengths, greater clustering, and higher eigenvector centrality, which normalises to healthy control levels in AD.

#### 3.2.2 Local dynamics

**Fig. 8.**
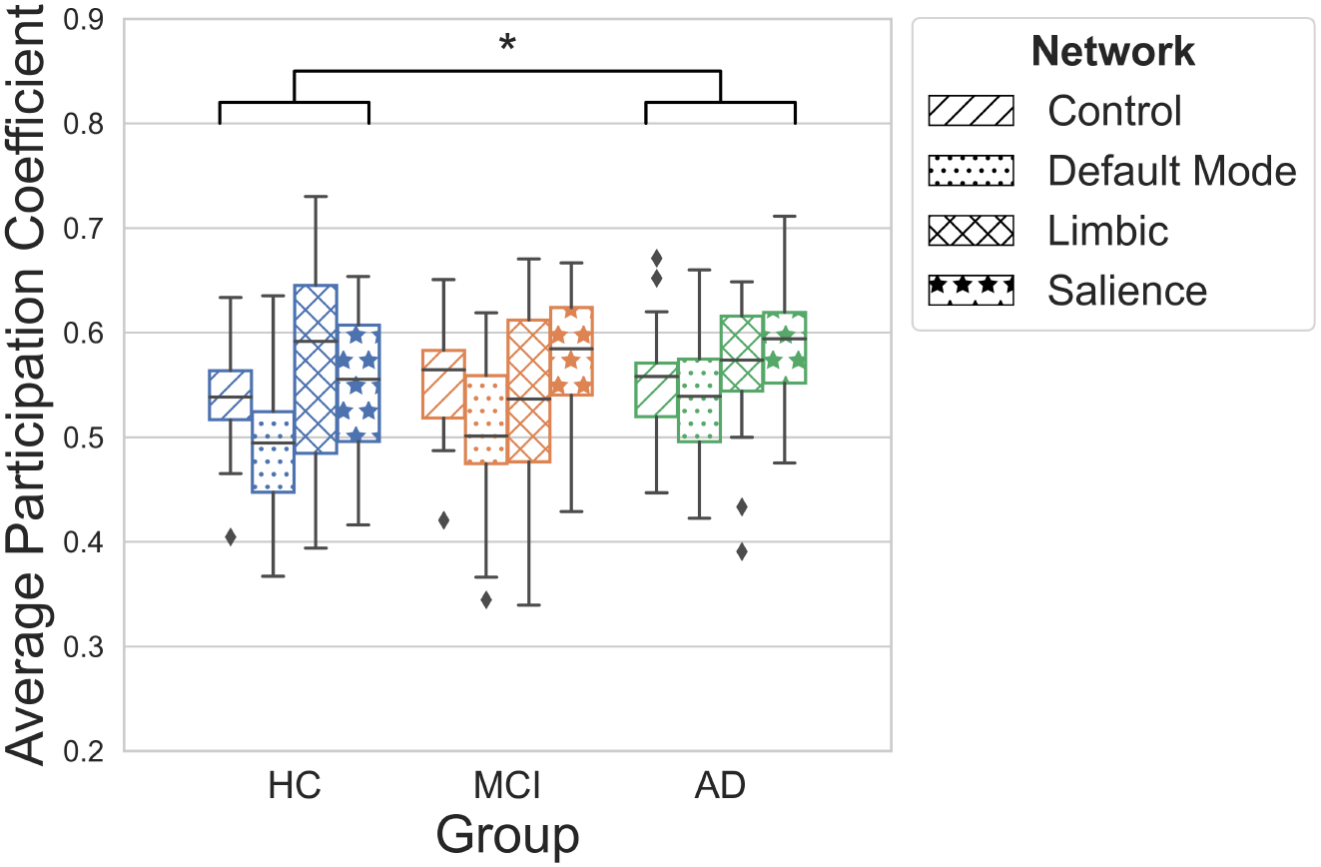
Average participation coefficient per network across groups, reported as the median with interquartile range. A two-way ANOVA found a main effect of group (*p* < 0.05) and network (*p* < 0.001); post hoc comparisons conducted using Tukey’s HSD test. Statistical significance denoted by *, p < 0.05; FDR corrected. Network differences not shown for visual clarity.

To identify specific networks which are highly connected, graph theory metrics were also calculated at the local level. Clustering properties were first considered. Modularity did not differ between groups (*H*(2) = 0.97, *p* = 0.62); however, a two-way ANOVA found a main effect of group (Fig. 8; *F*(2,240) = 6.05, *p* < .05, *η*^2^ = 0.044) and resting-state network (*F*(3,240) = 9.06, *p* < .001, *η*^2^ = 0.088) on participation coefficient. No interaction between group and network was observed (*F*(6,240) = 0.97, *p* = 0.45). Post hoc comparisons revealed overall significantly higher coefficients in AD than in healthy controls (*g* = 0.37, *p*_FDR_ < .05) indicating greater connectivity of parcels with those in clusters outside of their own. Additionally, lower coefficients in the DMN than in the control (*g* = -0.61, *p*_FDR_ < .05), limbic (*g* = -0.55, *p*_FDR_ < .01), and salience networks (*g* = -0.91, *p*_FDR_ < .001) were found across all groups.

**Fig. 9.**
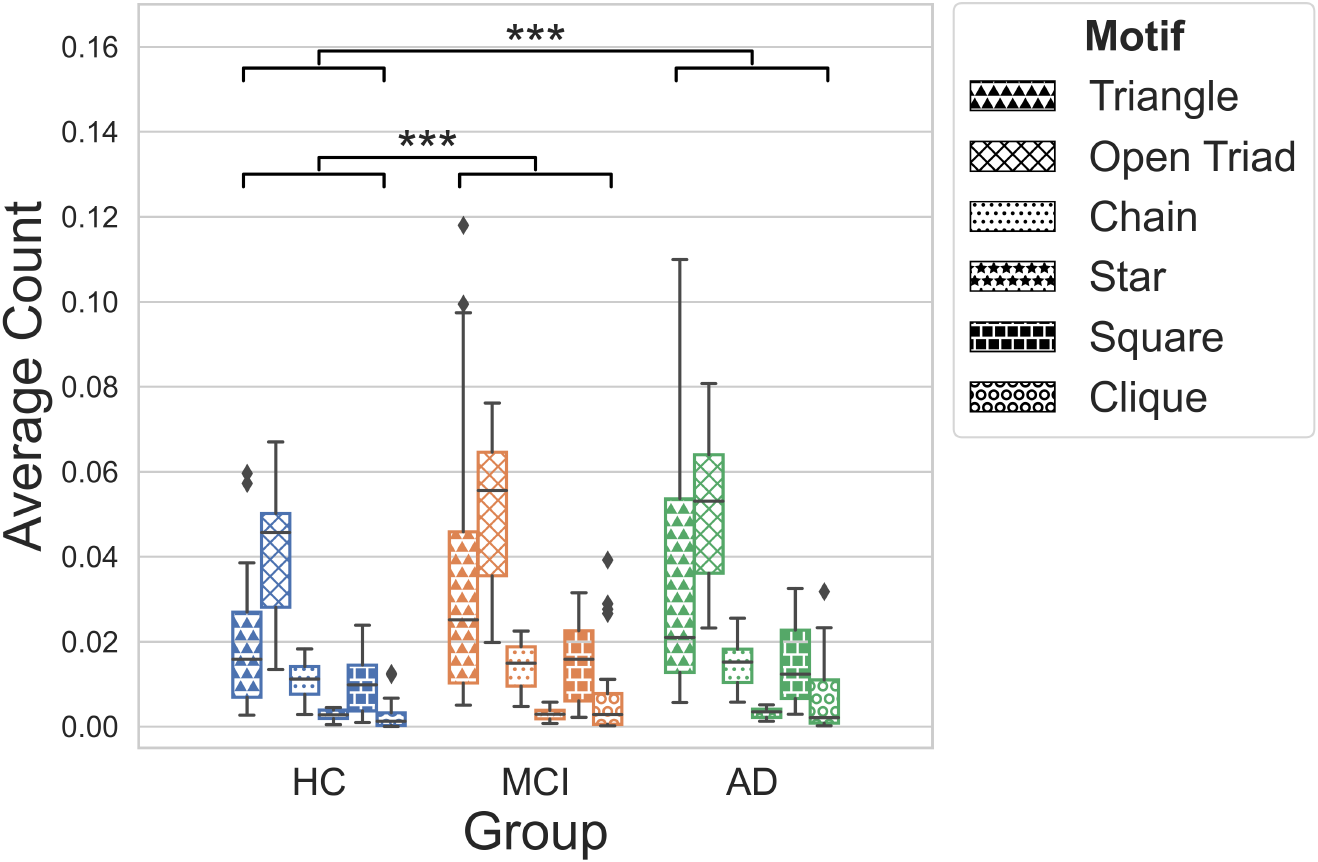
Average count of motif type across groups, reported as the median with interquartile range. An ART-ANOVA found a main effect of group (*p* < 0.001) and motif type (*p* < 0.001) on motif count; post hoc comparisons conducted using Tukey’s HSD test. Statistical significance denoted by ***, *p* < 0.001; FDR corrected. Motif differences not shown for visual clarity.

A graph can also exhibit various motifs, that is, distinct sub patterns of connectivity between nodes, which can provide insights into a graph’s functional organisation. The prevalence of different types of motifs was quantified and compared across groups (Fig. 9). An Aligned-Rank Transformation (ART) ANOVA revealed a main effect of group (*F*(2,441) = 12.63, *p*_FDR_ < .001) and motif type (*F*(6,441) = 266.97, *p*_FDR_ < .001) on the number of motifs present. No interaction between group and motif type was found (*F*(12,441) = 0.87, *p*_FDR_ = .58). Post hoc comparisons revealed a significantly higher prevalence of motifs in the AD (*g* = 0.29) and MCI groups (*g* = 0.31) than in healthy controls (*p*_FDR_ < .001). As this finding may simply reflect higher degree density, the ratio of triangles (indicating local clustering) to open triads (reflecting a less cohesive architecture) was calculated; however, no difference was found between groups (*H*(2) = 3.76, *p* = 0.16).

**Fig. 10.**
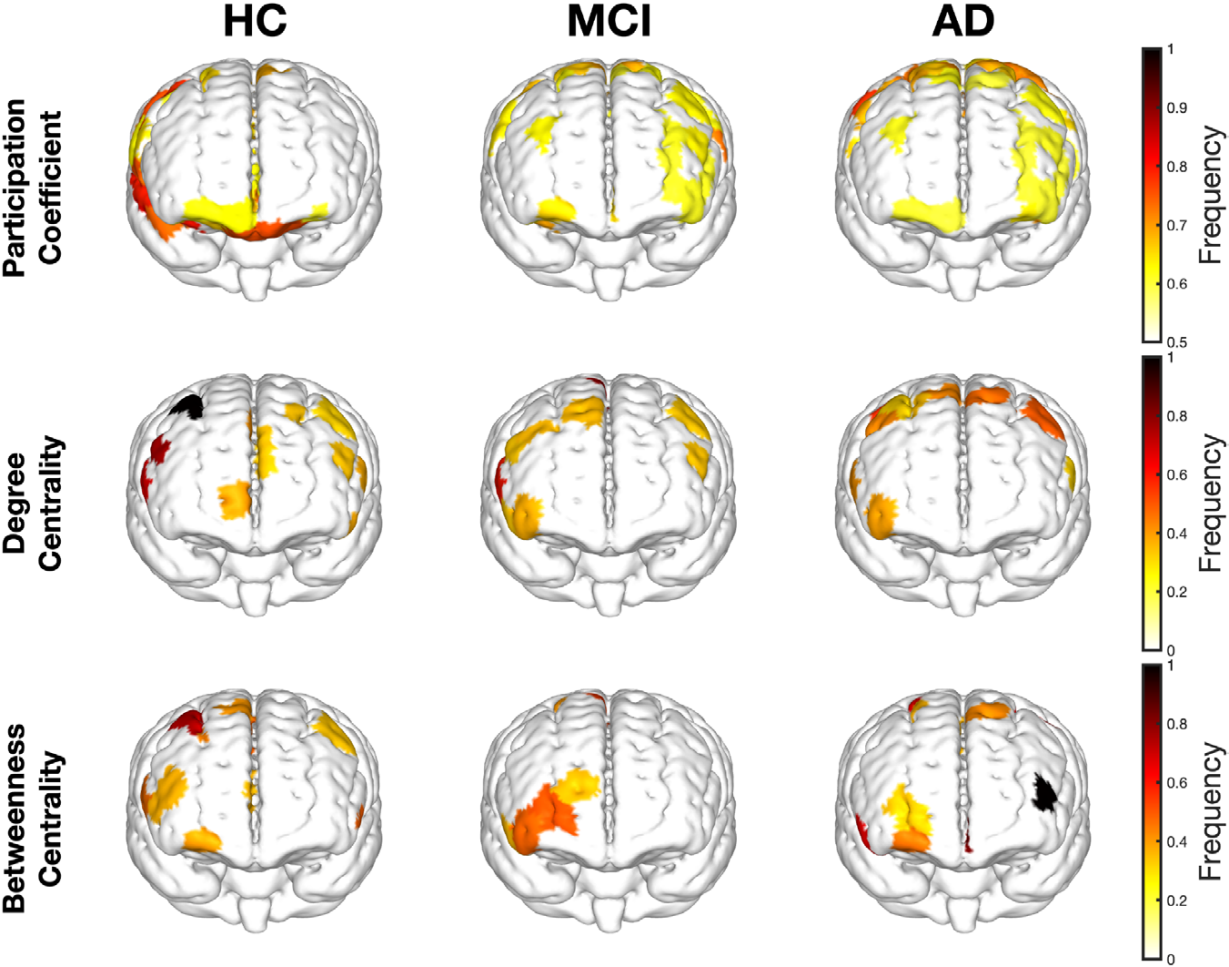
Normalised frequencies of high-scoring parcels per metric. Presented on a standard MNI atlas. Parcels defined as “high” scoring if present in the top 10% for each measure in at least 20% of subjects. Frequency of participation coefficients thresholded to only include frequencies > 0.6 for visual clarity.

To identify key network hubs within each group, parcels exhibiting consistently high functional connectivity across subjects were identified and quantified according to their normalised hub occurrence (Fig. 10). Healthy controls exhibited a small number of consistently-occurring core hubs (i.e., identified in over 50% of subjects), predominantly located within the control network and in the right hemisphere. These hubs were characterised by high clustering, efficiency, and eigenvector centrality, indicative of strong local integration and central network influence. In MCI, the hub profile was markedly altered. Although the total number of hubs was comparable with healthy controls (MCI: 60, HC: 56), consistently occurring hubs were re-distributed across control, salience/ventral attention, and default mode networks, and exhibited lower overall centrality (MCI: 0.37 [0.36–0.39], HC: 0.42 [0.36–0.47]). The most stable hubs in MCI were those defined by clustering and efficiency, with fewer hubs identified for degree, suggesting a predominance of locally connected, rather than globally connected hubs. For example, the lateral PFC within the DMN was identified as a hub for clustering and efficiency in 62.5% of subjects, but for degree in only 38%. In further contrast to healthy controls, hubs in MCI were distributed bilaterally across hemispheres. In AD, the re-distribution of hub-like properties was even more pronounced. Hubs were more broadly dispersed across dorsal attention, salience/ventral attention, and control networks, with greater variability in overall centrality than MCI and healthy controls (AD: 0.40 [0.32–0.50]). Similar to MCI, the majority of hubs were consistently identified based on clustering and efficiency (63% and 75% of subjects, respectively), whereas fewer hubs were identified based on degree (15%) of subjects, reflecting a shift toward locally connected rather than globally connected network organisation.

#### 3.2.3 Association with clinical data

**Fig. 11.**
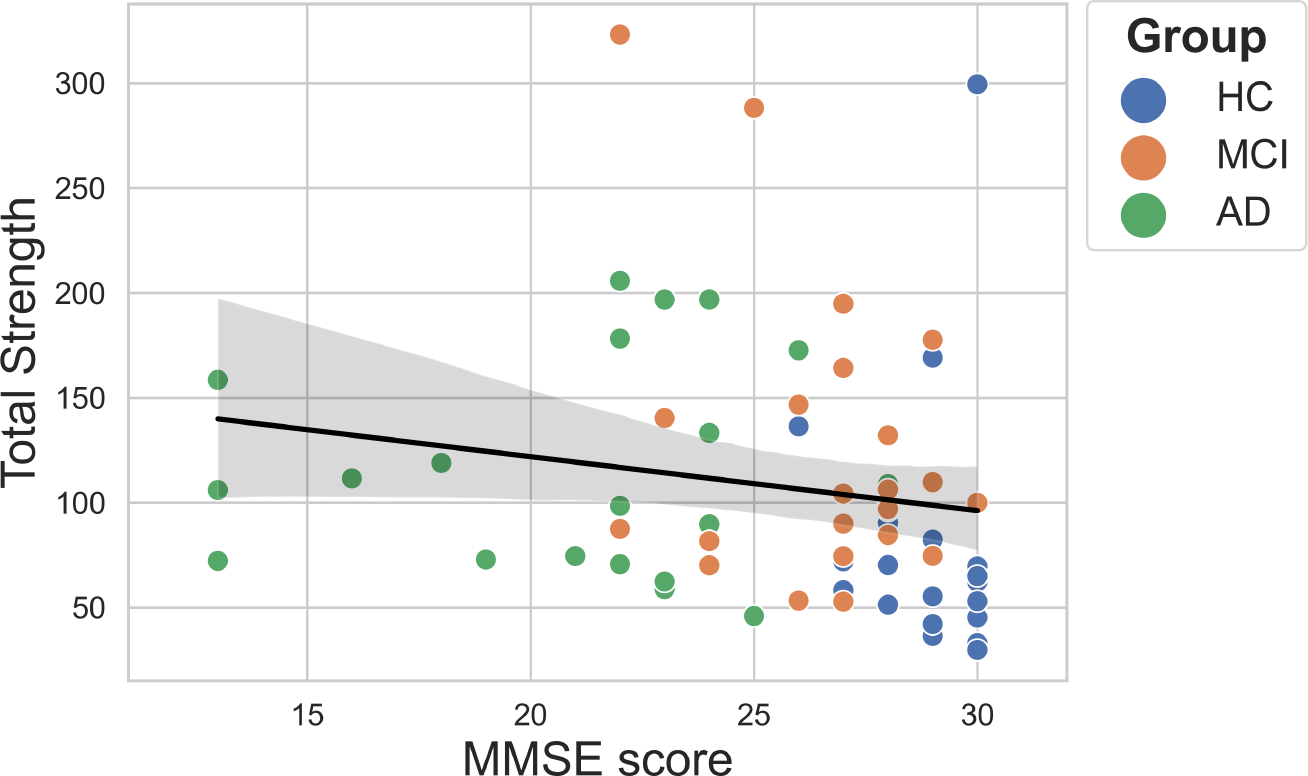
Correlations between total strength and Mini-Mental State Examination score (*p* values corrected using FDR).

There was no association between any of the global metrics and either MTA rating or age when considering all groups together. A weak correlation was found between MMSE score and total strength (*r* = -0.32, *p* < .01); however, this was not significant when tested within groups (Fig. 11). As total strength scales with the number of parcels present, partial correlations controlling for parcel count were computed. Following this correction, total strength was not significantly associated with MMSE score.

## 4 Discussion

Dementia has been described as a “disconnection syndrome”, wherein large-scale disruptions in functional connectivity are clinically relevant: they are thought to emerge decades before symptom onset (Manno et al., 2019; Pereira, 2020), respond to medication (Lorenzi et al., 2011; Rizzi et al., 2022), are observed in mouse models (Zott et al., 2019), and mirror the spread of AD pathology (Griffa et al., 2013; Palmqvist et al., 2017). In this study, we applied a graph theory approach to quantify prefrontal resting-state functional connectivity, as measured by HD-DOT, in individuals with AD-type MCI, AD-type dementia, and in age-matched healthy controls, which to our knowledge has not been previously explored. Such network-based approaches facilitate cross-study comparisons and provide insights into how dynamic properties emerge from underlying network topology. We identified group differences in prefrontal network organisation at both global and local levels, alongside distinct profiles of key network hubs characterising each group. Consistent with our hypotheses, network organisation differed across groups; however, the direction of these changes varied by disease stage. While we expected progressive disruption of functional connectivity in AD with less pronounced alterations in MCI, we instead observed marked hyperconnectivity in MCI, characterised by increased density, clustering, eigenvector centrality, and efficiency relative to controls and AD. The combination of shorter pathlengths, increased density, and higher total strength suggests that networks in MCI are more globally connected albeit in a less coordinated manner, consistent with disrupted metastability (Córdova-Palomera et al., 2017; Penalba-Sánchez et al., 2023). The redistribution of key network hubs in MCI, accompanied by reduced centrality, further indicates altered network organisation that may reflect early destabilisation or compensatory recruitment of additional neural resources. Although prefrontal connectivity largely normalised to healthy control levels in AD, network hubs became further dispersed and were associated with graph-theoretical measures that indicated a general shift toward locally connected rather than globally integrated networks. Taken together, these findings suggest that graph-theoretical analysis of prefrontal resting-state HD-DOT is sensitive to functional network reorganisation associated with declining function and accumulating pathology in AD-type cognitive impairment.

Changes in connectivity can reflect both maladaptive and compensatory mechanisms, so the functional significance of the observed prefrontal hyperconnectivity in MCI is unclear. According to the dual-stage model of AD progression (Finn et al., 2015), early hyperconnectivity—driven by disease and gene-related factors—is thought to be compensatory, arising in response to declining MTL-PFC connectivity (Berron et al., 2020). In this model, the brain initially attempts to recruit alternative pathways to preserve cognitive function in the short term whereby increased local connectivity supports communication near sites of damage, while increased global connectivity reflects the engagement of additional network hubs to maintain overall network function (Penalba-Sánchez et al., 2023). At this stage, regions not yet burdened by tau pathology exhibit an imbalance in neuronal excitability, characterised by reduced inhibitory GABAergic activity and increased excitatory glutamatergic activity. There is growing preclinical and modelling evidence that such heightened neuronal excitability may promote amyloid-*β* deposition and facilitate tau pathology (Barbour et al., 2025; Bero et al., 2011; Nishida et al., 2024; Sanchez-Rodriguez et al., 2024), consistent with the “activity causes damage” hypothesis whereby regions previously acting as hubs experience heavy pathology and lose their hub function (Targa Dias Anastacio et al., 2022). Thus while early hyperconnectivity may be initially adaptive, it can ultimately exacerbate disease progression by increasing vulnerability to tau and amyloid deposition (Bonanni et al., 2021). The presently-observed increased prefrontal connectivity in MCI aligns with this framework wherein connectivity subsequently reduces as pathology accumulates, white matter tracts degenerate, and synapses are lost, ultimately disrupting communication between brain regions (Mijalkov et al., 2023). This interpretation is supported by the observed redistribution of network hubs and accompanying higher variability in centrality measures in AD compared to both MCI and healthy controls.

Another possibility is that systemic physiological components contaminated the cortical signal, such that vascular, rather than purely neural, contributions to the present findings cannot be excluded. Prior work has demonstrated that even after regressing extra-cerebral signals captured by short channels from the cortical signals of longer channels, residual systemic influences can still artificially inflate functional connectivity estimates calculated using NIRS (Abdalmalak et al., 2022). Similarly, controlling for cardiovascular factors has been shown to eliminate age-related effects on resting-state amplitude fluctuations measured with fMRI (Tsvetanov et al., 2020). In the present study, potential vascular confounds were addressed by regressing the physiological signal from the nearest short channel onto each long channel, in line with current best practices in HD-DOT (Brigadoi & Cooper, 2015; Wyser et al., 2020), and by performing global signal regression to further reduce physiological contamination as is recommended in both fMRI (e.g., Li et al., 2019) and NIRS (e.g., Lanka et al., 2022). Although global signal regression can introduce artefacts and distort connectivity estimates (Gotts et al., 2013; Murphy et al., 2008), the observed correlation between the global component and time series was higher in MCI and AD than in healthy controls suggesting that underlying vascular alterations may have biased the findings.

While there is no prior work applying HD-DOT to dementia, previous studies using NIRS have indeed identified alterations in functional connectivity in MCI and AD, though there is no clear consensus on the nature of these changes. This is in part owing to the limited number of studies published in the field and the heterogeneity in methodology across these studies (Albrecht et al., 2025; Bray et al., 2025). Findings are variable: both increased and decreased connectivity have been observed in MCI and AD (Butters et al., 2023). For example, some studies have reported reduced PFC connectivity in MCI (Ghafoor et al., 2019; Pu et al., 2025) and in AD (Keles et al., 2022) compared with controls, although these differences were not tested for significance. Additionally, reduced whole-brain connectivity (Zhang et al., 2022) and diminished effective connectivity between distal regions of the brain, for example, between the PFC and the occipital lobe, have been reported in MCI (Bu et al., 2019). Conversely, *increased* inter-hemispheric PFC connectivity in MCI (Nguyen et al., 2019) and higher prefrontal spectral entropy in AD (Ferdinando et al., 2023) have also been reported. Some studies, however, find no differences in connectivity (Yang et al., 2025; Zheng et al., 2023). In the only study directly comparing MCI and AD, no differences in whole-brain functional connectivity were found (Niu et al., 2019). It is challenging to compare the present results with these studies for a number of reasons, which are detailed in Albrecht et al. (2025). Many studies used low-density systems with a single optode per region of interest (e.g., Bu et al., 2019). While functional connectivity is generally more sensitive to diversity across regions than within the same region, the higher resolution in the present study allows for a more fine-grained representation of brain activity and improved delineation of brain networks. Additionally, no prior studies used subject-specific anatomical priors for signal reconstruction which may have led to the under-estimation of the haemodynamic signal. Moreover, while many studies measured functional connectivity using the average Pearson’s correlation coefficient between brain regions, graph theory offers a more comprehensive quantification of the *structural* organisation of the network. The only study to apply graph theory did so (Ghafoor et al., 2019) with a low-density system, perhaps lacking the spatial resolution necessary for detailed graph analysis.

Turning instead to the large body of work using fMRI, a general shift from early stage hyperconnectivity to later stage hypoconnectivity in AD-type dementia has been documented (albeit with substantial individual variability; Dickerson et al., 2005; Pini et al., 2025). Looking specifically to prefrontal connectivity, evidence is mixed for MCI but AD is more consistently associated with hypoconnectivity (Ibrahim et al., 2021), which was not observed in the present study across any of the graph theory metrics. It is uncertain whether connectivity measured by fMRI and HD-DOT captures the same underlying construct (Tijms et al., 2013). The HD-DOT signal primarily reflects local haemodynamic activity within the microvasculature which occurs in superficial cortical layers and may be affected in early stages of disease progression, whereas fMRI captures generally broader activity, including that from deeper brain regions (Sasai et al., 2012). Therefore, while the present results may reflect early disrupted prefrontal cortical connectivity, they do not exclude network disruption in temporal or parietal cortices, or disruption in deeper brain regions, as observed with fMRI (Li et al., 2014). A key finding across fMRI studies is reduced PFC–MTL coupling and selective vulnerability of long-range connections (Berron et al., 2020). However, these connections cannot be explored using the current HD-DOT data due to limited field-of-view and depth penetration, as the MTL is not imageable by NIRS. While there is value in focusing on the PFC, as it is thought to be crucial in maintaining cognitive function during neurodegeneration (Jobson et al., 2021), doing so paints a limited picture of the complex interplay between the brain areas affected in dementia. The MTL atrophy rating scale used to rate hippocampal atrophy in the present study also may not serve as a relevant marker of structural change that can be directly compared with the functional changes observed in the PFC as the MTL and PFC differ in the timeline of their structural and functional changes (Jobson et al., 2021).

Distinct alterations between AD and MCI have also been identified across several large-scale functional brain networks (Li et al., 2014). One network of particular interest, the DMN, has been well characterised using fMRI. This network is typically more active during rest and deactivates during task states (Raichle et al., 2001). Altered connectivity within the DMN has been widely reported in AD (Ereira et al., 2024; Toussaint et al., 2014) and has shown strong potential for diagnosis (Ibrahim et al., 2021), as it is the first network affected by amyloid deposition (Buckner et al., 2009; Mintun et al., 2006). Although DMN connectivity was not captured in the present study, the lateral PFC—part of the DMN—emerged as an important network hub in MCI. It has been proposed that key hubs in the DMN may be selectively vulnerable in AD, as signal fluctuations in the DMN have been found to negatively correlate with amyloid burden (Scheel et al., 2021). Furthermore, in AD and MCI, key functional hubs were located bilaterally, whereas in controls, hub activity was largely confined to the right hemisphere. Such changes in frontal lateralisation have been observed in both NIRS and (Gjonaj et al., 2025) and fMRI (Liu et al., 2018), suggesting functional brain reorganisation in AD-type cognitive impairment.

There is a growing push to standardise dementia stratification using biologically-based diagnostic criteria. For example, the ATN framework for AD classifies individuals according to objective markers of amyloid, tau, and neurodegeneration (Jack et al., 2016). The inclusion of a marker of brain *function* (Finn et al., 2015) into such classifications remains a topic of debate. To this end, functional connectivity may capture distinct clinical phenotypes present early in the disease course, which are not captured by traditional markers such as structural atrophy (Pini et al., 2025). Yet incorporating such a functional marker would only be possible using tools which are accessible and scalable using current infrastructures. The present work demonstrates the potential of HD-DOT for this purpose. The majority of data collection took place in the home setting and we found no difference between the quality of the data collected in the home and that collected in a for-purpose clinical room at the University of Cambridge. The practical advantages of HD-DOT make it particularly well suited for scalable clinical use, facilitating widespread deployment both in settings outside of the clinic and at multiple time points to support personalised treatment and diagnostic approaches. Nevertheless, analysis pipelines for HD-DOT remain largely unstandardised (Yücel et al., 2021) and current methods rely on computationally expensive and inaccessible techniques such as photogrammetry and MRI, which curtails its practicality. Efforts can be made to overcome these challenges though. While assuming a standard brain size and shape likely introduces error (Srinivasan et al., 2023), as has been done in all previous work applying NIRS in dementia, acquiring a subject-specific MRI is often impractical. For such cases, an *atlas* for various dementia subtypes could be developed, as has been done for infants (Collins-Jones et al., 2021). An appropriate head model could be chosen based on various parameters such as head size, age, and symptom history.

### 4.1 Limitations

The present study has certain limitations. Firstly, we used what is considered to be a relatively short resting-state duration. Typically, the optimal acquisition time for resting-state fMRI is around 10 minutes, as test–retest reliability of functional connectivity measures improves with longer scan durations (Birn et al., 2013). However, this should be considered in the context of fMRI’s lower sampling rate of around 0.5–1 Hz, compared with the generally higher sampling rates of NIRS and HD-DOT, and that of 12.5 Hz used in the present study. In fact, previous work using NIRS has shown that as little as 30 seconds of resting-state data can distinguish between MCI and AD (Yang & Hong, 2021). Secondly, as the HD-DOT signal is a multiplexed combination of neural, haemodynamic, and noise components, entirely disentangling vascular and neural contributions to the signal are not possible (Ereira et al., 2024). This is particularly pertinent to dementia where we expect underlying disease-related vascular changes to influence cerebral haemodynamics (de la Torre, 2012). While efforts were made to minimise the influence of systemic physiology on the signal, including short-channel, and global signal regression, the degree to which the presently observed differences in functional connectivity reflect true neural activity is unknown. This issue is not unique to HD-DOT however, it is also inherent to fMRI (Drew, 2019), and can be further addressed by the use of simultaneous EEG-NIRS paradigms.

### 4.2 Future work

To our knowledge, this is the first study that has applied HD-DOT to any type of dementia, opening up several avenues for future research in the area. Dementia leads to widespread dysfunction across many cortical and sub-cortical brain regions (Raji et al., 2009). Evaluating whole-brain functional connectivity would, therefore, help to provide a more comprehensive understanding of the patterns in the prefrontal cortex observed in the present work. This could be achieved using newly-developed whole-head HD-DOT systems (Collins-Jones et al., 2024). In addition, using task-based data instead of the resting state may tease out more pronounced differences between groups and reveal more subtle within-group variations (Nguyen et al., 2019). The “Optical Neuroimaging and Cognition” study employed a range of task paradigms, the data of which remain to be explored, and also used broadband NIRS to assess neurometabolism (Acharya et al., 2025). Finally, alternative statistical methods could be applied to our data to provide further insights into networks beyond their structural organisation. For example, computing effective connectivity would determine the directionality of functional relationships between brain regions (Ereira et al., 2024). A more mechanistic approach would be dynamic causal modelling (Tak et al., 2015), which explicitly models the processes underlying these relationships. This type of modelling has not yet been applied to HD-DOT as far as we are aware.

## 5 Conclusion

In summary, we have shown that HD-DOT is capable of detecting differences in both local and global prefrontal functional connectivity in the resting state between individuals with AD-type dementia and AD-type MCI, and healthy controls. These findings suggest increased network density and strength in AD and MCI, alongside a loss of hierarchical organisation and key network hubs as the disease progresses. Through this work, we have also demonstrated the feasibility of using HD-DOT for high-quality, at-home assessments of brain function. Future work should employ longitudinal study designs to assess the sensitivity of HD-DOT to disease-related changes, and should prioritise the development of standardised analysis and preprocessing pipelines to improve the useability of HD-DOT in clinical settings.

## Supplementary Material

Supplementary Material

## Figures and Tables

**Appendix Table A1.** MRI sequence parameters across studies.

Study	Scanner	TE (ms)	TR (ms)	Flip angle (°)	Slice thickness (mm)
ONAC	3T Siemens MAGNETOM Prismafit	2.98	2300	9	1.0
	(Siemens; Hamburg, Germany)				
MILOS	3T Siemens MAGNETOM Prismafit	2.98	2300	9	1.0
	(Siemens; Hamburg, Germany)				
7T	7T Siemens Terra	1.99	4300	5/6	0.75
	(Siemens; Hamburg, Germany)				
QMIN-MC	3T Siemens MAGNETOM Prismafit (Siemens; Hamburg, Germany)	2.98	2000	9	1.0

## Data Availability

The code is available at www.github.com/emiliavioletb/ImagingNeuroscience. Data are available upon reasonable request from accredited researchers, in accordance with our ethical approval.

## Appendix

### A.2. Details of MRI acquisition

Across the MCI and AD groups, a total of 43 subjects required MRI scans for the creation of head models. Of these, 26 were scanned through the “Optical Neuroimaging and Cognition” study, 4 through the “MultiModal Imaging in Lewy-Body Disorders” study, 4 through the “7T MRI for Dementia with Lewy Bodies”, and 9 through the “Quantitative MRI in the NHS Memory Clinics” study. The respective sequence parameters are detailed in Appendix Table A1.

Scans acquired at 7T were de-noised using a custom script (www.github.com/benoitberanger/mp2rage) and bias field corrected using SPM12 (www.fil.ion.ucl.ac.uk/spm) with light regularisation (0.001).

### A.3. Optode array coverage

There were no differences in the number of sensitive parcels across groups (*H*(2) = 2.10, *p* = 0.35), as shown in Appendix Figure A2a.
