# Supplementary material for: Brain network analysis in Alzheimer’s disease and mild cognitive impairment using high-density diffuse optical tomography
Source: Imaging Neurosci (Camb). 2026 Apr 24;4:IMAG.a.1208. doi: 10.1162/IMAG.a.1208 (PMC13112212; doi:10.1162/IMAG.a.1208)
Supplement: Supplementary Material [file IMAG.a.1208_supp.pdf]

Supplementary material

Table S1: Summary of neuropsychological test scores across study groups.

	**HC** (*n*=22)	**AD** (*n*=21)	**MCI** (*n*=22)	**p**
Attention/processing speed				
Digit span	9.77 ± 2.04	5.43 ± 2.50	8.19 ± 1.74	**0.00**[a,b,c]
TMT-A time (s)	41.6 ± 12.9	117 ± 87.0	50.8 ± 21.3	**0.01**[b,c]
TMT-A errors	0.09 ± 0.29	0.33 ± 1.08	0.20 ± 0.6	0.76
Executive function				
Interference sensitivity	2.91 ± 0.29	1.81 ± 1.26	2.95 ± 0.21	**0.00**[b,c]
Inhibitory control	2.64 ± 0.71	1.29 ± 1.12	2.38 ± 1.05	**0.01**[b,c]
TMT-B time (s)	85.1 ± 41.6	54.2 ± 93.7	114 ± 46.2	**0.02**[b,c]
TMT-B errors	15.3 ± 2.22	1.19 ± 2.59	2.00 ± 2.60	0.45
Motor function				
UPDRS Part III	3.14 ± 3.11	7.67 ± 7.14	4.82 ± 3.95	0.37
Olfactory function				
B-SIT	9.09 ± 2.64	6.10 ± 2.79	7.65 ± 2.89	0.26
Colour discrimination				
Farnsworth total error	8.63 ± 18.2	20.2 ± 21.4	6.84 ± 9.54	**0.04**[c]
Visual hallucinations				
Pareidolia score	37.7 ± 8.44	34.7 ± 8.87	39.5 ± 0.84	**0.01**[b,c]
Presence of hallucinations (%)	0	9.50	9.10	0.98
Anxiety & depression				
HADS	5.18 ± 4.53	7.33 ± 3.63	9.58 ± 4.50	0.37
GDS	1.50 ± 2.79	2.43 ± 2.13	3.64 ± 3.38	0.37
Informant questionnaires				
BADLS	n/a	9.95 ± 7.02	2.50 ± 3.61	**0.00**
CDR	n/a	6.91 ± 3.43	4.06 ± 2.72	**0.01**
CBI	n/a	42.0 ± 17.6	26.6 ± 14.0	**0.01**
CAF	n/a	1.19 ± 2.56	3.50 ± 1.66	0.50
CAF (One day)	n/a	2.48 ± 2.84	0.55 ± 1.24	**0.01**
DCFS	n/a	9.71 ± 3.12	9.31 ± 2.20	0.37
NPI	n/a	18.2 ± 6.74	9.82 ± 10.3	0.07

Shown as mean ± standard deviation or percentage (%).

P-value indicates overall group-level comparison. Superscript letters denote significant pairwise group comparisons: [a] HC vs MCI, [b] HC vs AD, [c] MCI vs AD.

MMSE, Mini-Mental State Examination (Folstein et al., 1975); MoCA, Montreal Cognitive Assessment (Nasreddine et al., 2005); TMT, Trail Making Test (Partington & Leiter, 1949); UPDRS, Unified Parkinson's Disease Rating Scale (Goetz et al., 2008); B-SIT, Brief Smell Identification Test (Doty et al., 1996); Farnsworth; D-15 color arrangement test. Pareidolia; the Noise Pareidolia test (Mamiya et al., 2016); HADS, Hospital Anxiety and Depression scale (Zigmond & Snaith, 1983); GDS, Geriatric Depression Scale (Yesavage & Sheikh, 1986); BADLS, Bristol Activities of Daily Living Scale (Bucks et al., 1996); CDR, Clinical Dementia Rating (Morris, 1993); CBI, Cambridge Behavioural Inventory (Wear et al., 2008); CAF, Clinician Assessment of Fluctuation (Walker et al., 2000); DCFS, Dementia Cognitive Fluctuation Scale (Lee et al., 2014). NPI, Neuropsychiatric inventory (Cummings et al., 1994).

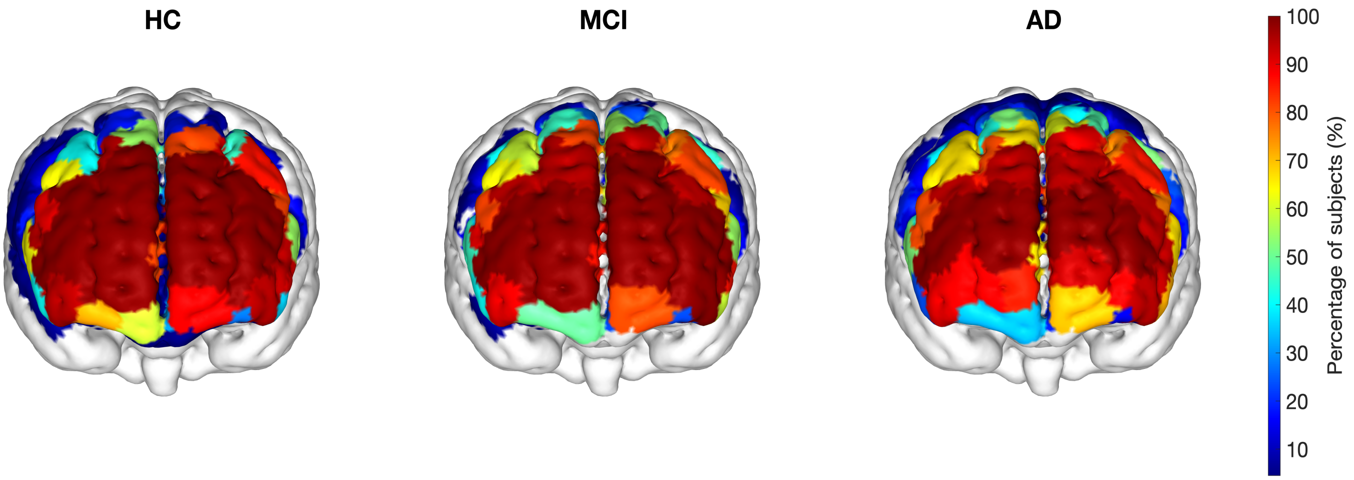

Figure S1: Histogram of sensitive parcels for each group, shown as a percentage. n = 65.

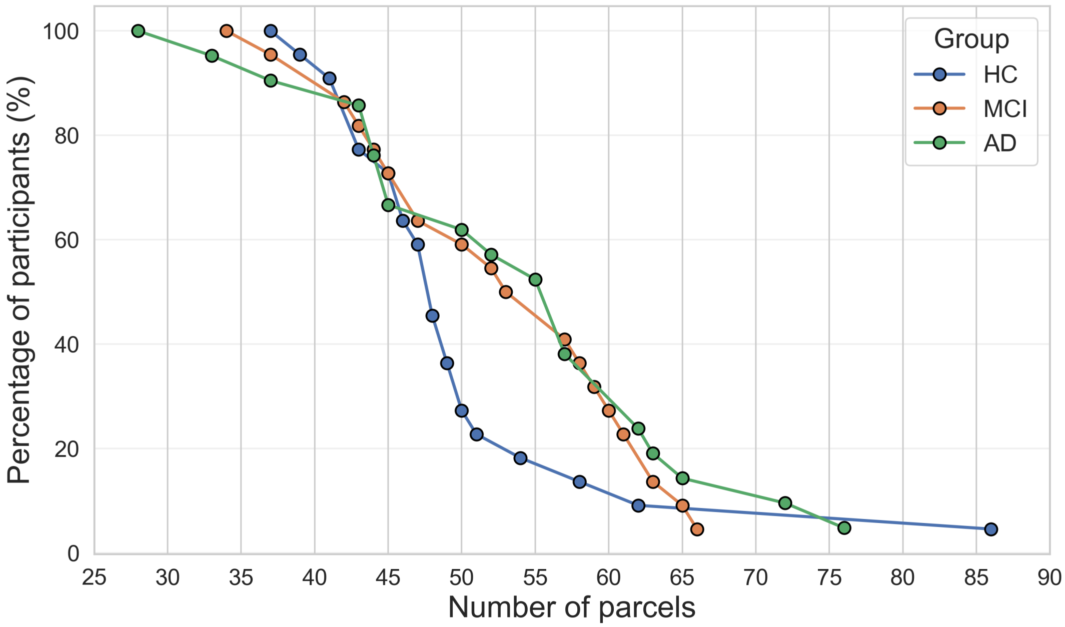

Figure S2: The percentage of participants as a function of the number of sensitive parcels within each group.

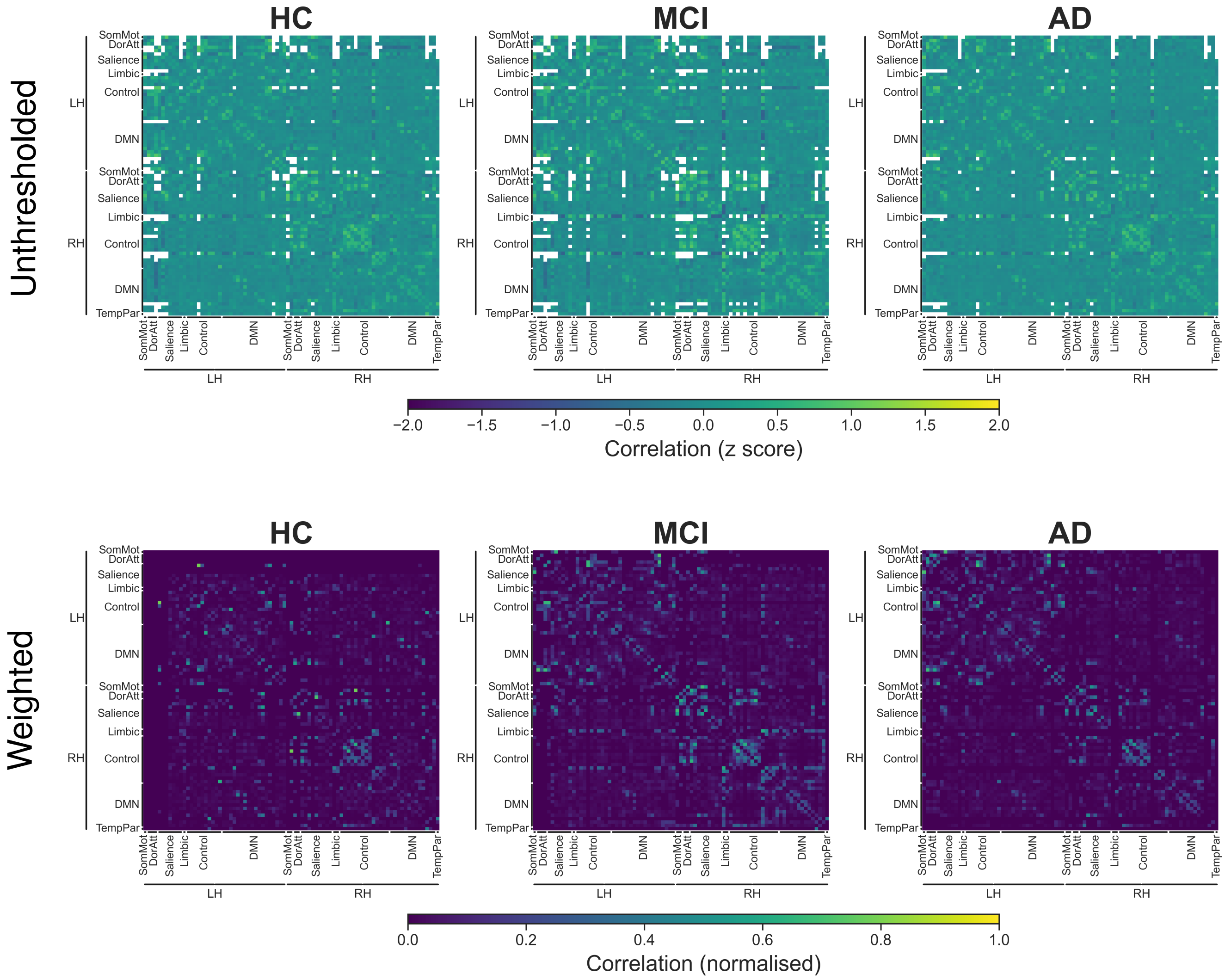

Figure S3: Group averaged unthresholded connectivity matrices (top) and weighted adjacency matrices (bottom) $< .05$) based on Fisher's z-transformed correlations. Grouped according to Yeo (2011) resting state network. All parcels identified as sensitive in any subject is shown.

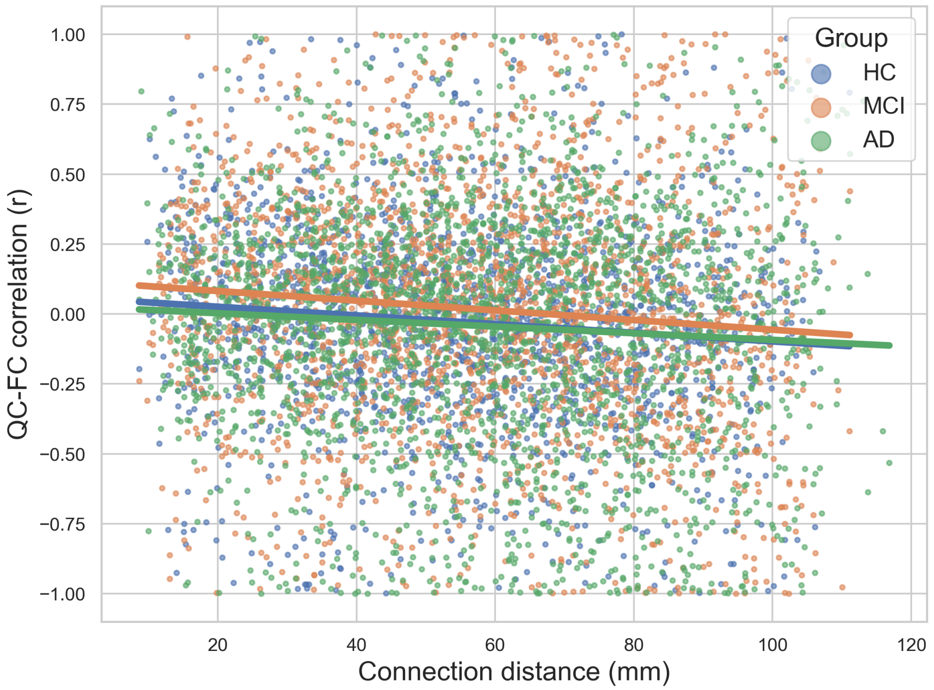

Figure S4: Quality Control (QC) – Functional Connectivity (FC) correlations as a function of connection distance per group. Each point represents an edge, plotted as the correlation between motion burden (% of recording affected by motion) and functional connectivity versus the Euclidean distance between parcels. QC – FC values were distributed around zero across connection distances for all groups (HC: r = -0.10, MCI: r = -0.10, AD: r = -0.07, $p < .001$, $r^2 \leq 0.01$), suggesting minimal distance-dependent motion bias in the dataset (Ciric et al., 2018).

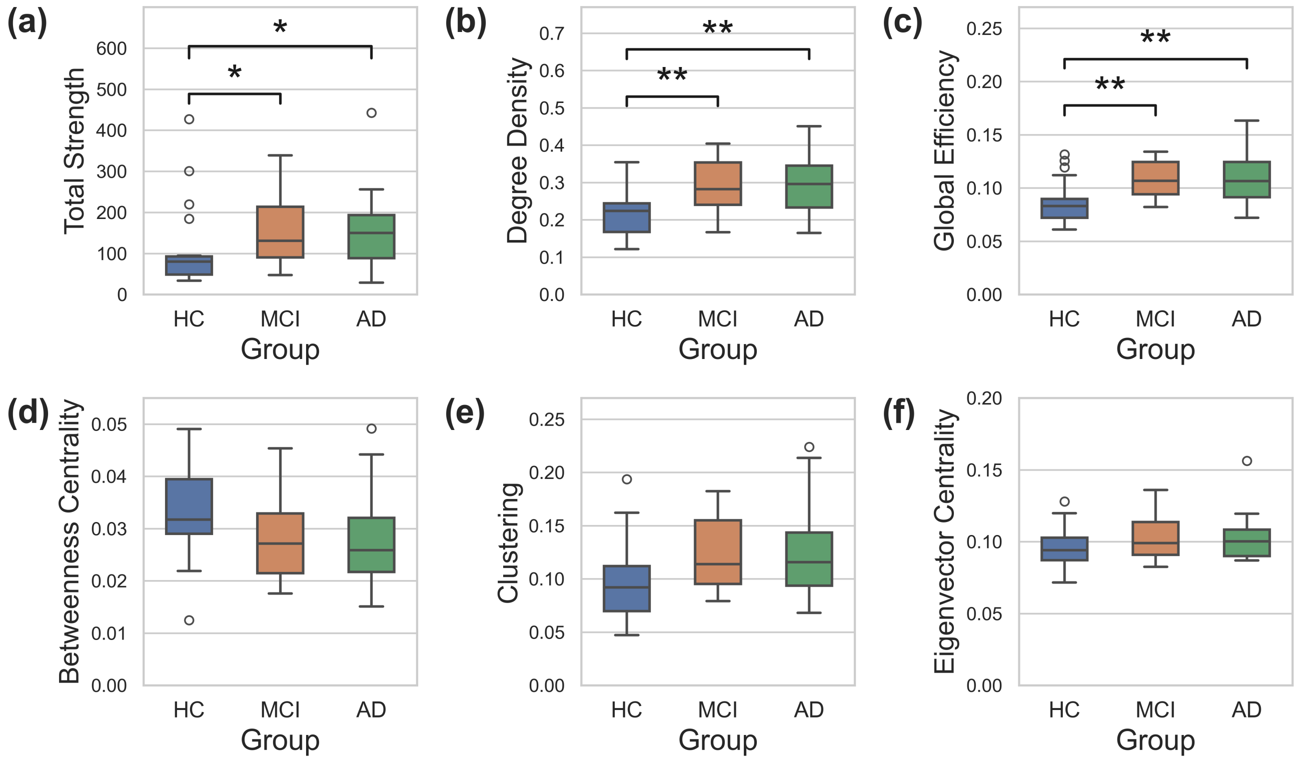

Figure S5: Group-level global functional connectivity without global signal regression, reported as the median with interquartile range. Statistical significance demonstrated by *, $p < 0.05$; **, $p < 0.01$; FDR corrected.

References

Bucks, R. S., Ashworth, D., Wilcock, G., & Siegfried, K. (1996). Assessment of activities of daily living in dementia: Development of the bristol activities of daily living scale. Age and ageing, 25(2), 113–120. https://doi.org/10.1093/ageing/25.2.113

Ciric, R., Rosen, A. F. G., Erus, G., Cieslak, M., Adebimpe, A., Cook, P. A., Bassett, D. S., Davatzikos, C., Wolf, D. H., & Satterthwaite, T. D. (2018). Mitigating head motion artifact in functional connectivity MRI. Nat Protoc, 13(12), 2801–2826. https://doi.org/10.1038/s41596-018-0065-y

Cummings, J. L., Mega, M., Gray, K., Rosenberg-Thompson, S., Carusi, D. A., & Gornbein, J. (1994). The neuropsychiatric inventory: Comprehensive assessment of psychopathology in dementia. Neurology, 44(12), 2308–2308. https://doi.org/10.1212/wnl.44.12.2308

Doty, R. L., Marcus, A., & William Lee, W. (1996). Development of the 12-item cross-cultural smell identification test (cc-sit). The Laryngoscope, 106(3), 353–356. https://doi.org/10.1097/00005537-199603000-00021

Folstein, M. F., Folstein, S. E., & McHugh, P. R. (1975). “mini-mental state”: A practical method for grading the cognitive state of patients for the clinician. Journal of psychiatric research, 12(3), 189–198. https://doi.org/10.1016/0022-3956(75)90026-6

Goetz, C. G., Tilley, B. C., Shaftman, S. R., Stebbins, G. T., Fahn, S., Martinez-Martin, P., Poewe, W., Sampaio, C., Stern, M. B., Dodel, R., et al. (2008). Movement disorder society-sponsored revision of the unified parkinson’s disease rating scale (mds-updrs): Scale presentation and clinimetric testing results. Movement disorders: official journal of the Movement Disorder Society, 23(15), 2129–2170. https://doi.org/10.1002/mds.22340

Lee, D. R., McKeith, I., Mosimann, U., Ghosh-Nodial, A., Grayson, L., Wilson, B., & Thomas, A. J. (2014). The dementia cognitive fluctuation scale, a new psychometric test for clinicians to identify cognitive fluctuations in people with dementia. The American journal of geriatric psychiatry, 22(9), 926–935. https://doi.org/10.1016/j.jagp.2013.01.072

Mamiya, Y., Nishio, Y., Watanabe, H., Yokoi, K., Uchiyama, M., Baba, T., Iizuka, O., Kanno, S., Kamimura, N., Kazui, H., et al. (2016). The pareidolia test: A simple neuropsychological test measuring visual hallucination-like illusions. PLoS One, 11(5), e0154713. https://doi.org/10.1371/journal.pone.0154713

Morris, J. C. (1993). The clinical dementia rating (cdr) current version and scoring rules. Neurology, 43(11), 2412–2412. https://doi.org/10.1212/wnl.43.11.2412-a

Nasreddine, Z. S., Phillips, N. A., Bédirian, V., Charbonneau, S., Whitehead, V., Collin, I., Cummings, J. L., & Chertkow, H. (2005). The montreal cognitive assessment, moca: A brief screening tool for mild cognitive impairment. Journal of the American Geriatrics Society, 53(4), 695–699. https://doi.org/10.1111/j.1532-5415.2005.53221.x

Partington, J. E., & Leiter, R. G. (1949). Partington's pathways test. Psychological Service Center Journal, 1, 9.

Walker, M., Ayre, G., Cummings, J., Wesnes, K., McKeith, I., O'brien, J., & Ballard, C. (2000). The clinician assessment of fluctuation and the one day fluctuation assessment scale: Two methods to assess fluctuating confusion in dementia. The British Journal of Psychiatry, 177(3), 252–256. https://doi.org/10.1192/bjp.177.3.252

Wear, H. J., Wedderburn, C. J., Mioshi, E., Williams-Gray, C. H., Mason, S. L., Barker, R. A., & Hodges, J. R. (2008). The cambridge behavioural inventory revised. Dementia & neuropsychologia, 2, 102–107. https://doi.org/10.1590/S1980-57642009DN20200005

Yeo, B. T. T., Krienen, F. M., Sepulcre, J., Sabuncu, M. R., Lashkari, D., Hollinshead, M., Roffman, J. L., Smoller, J. W., Zöllei, L., Polimeni, J. R., Fischl, B., Liu, H., & Buckner, R. L. (2011). The organization of the human cerebral cortex estimated by intrinsic functional connectivity. J Neurophysiol, 106(3), 1125–1165. https://doi.org/10.1152/jn.00338.2011

Yesavage, J. A., & Sheikh, J. I. (1986). 9/geriatric depression scale (gds) recent evidence and development of a shorter version. Clinical gerontologist, 5(1-2), 165–173. https://doi.org/10.1300/J018v05n01_09

Zigmond, A. S., & Snaith, R. P. (1983). The hospital anxiety and depression scale. Acta psychiatrica scandinavica, 67(6), 361–370. https://doi.org/10.1111/j.1600-0447.1983.tb09716.x
